# Supplementary material for: Adjuvant Lipoic acid Injection in Sepsis treatment in China (ALIS study): protocol for a randomised, single-blind, placebo-controlled trial
Source: BMJ Open. 2023 Jul 30;13(7):e072897. doi: 10.1136/bmjopen-2023-072897 (PMC10387639; doi:10.1136/bmjopen-2023-072897)
Supplement: Supplementary data [file bmjopen-2023-072897supp002.pdf]

Supplementary Table 1 Trial schedule of the LAS study

Timepoint	Baseline	[a]Interventions								Termination	Follow-up period
	[b]D0	D1	D2	D3	D4	D5	D6	D7	D8-10	D…	D28	D…
Eligibility screening	×											
Informed consent obtaining	×											
Randomization	×											
Medical history collection	×											
Population data collection	×											
Baseline data collection	×											
Formulation of drug administration plan	×											
Evaluation of drug-related adverse reactions		×	×	×	×	×	×	×	×			
Evaluate serious adverse events		×	×	×	×	×	×	×	×			

**Timepoint**	**Baseline**	**[a]Interventions**									**Termination**	**Follow-up period**
	[b]D0	D1	D2	D3	D4	D5	D6	D7	D8-10	D…	D28	D…
Vital signs	×	×	×	×	×	×	×	×			×	
SOFA/APACHEIIscore	×	×		×		×		×				
[c]Need and duration of life support treatment		×	×	×	×	×	×	×	×	×	×	
[d]Levels of inflammatory mediators	×	×		×		×		×				
Arterial lactic acid level	×	×		×		×		×				
Transfer out of ICU		×	×	×	×	×	×	×	×	×	×	×
ICU death time		×	×	×	×	×	×	×	×	×	×	×
AKI occurrence time		×	×	×	×	×	×	×	×	×	×	×
Hospital death time		×	×	×	×	×	×	×	×	×	×	×
Discharge time		×	×	×	×	×	×	×	×	×	×	×

Eligibility screening includes diagnosis, inclusion/exclusion criteria, urine pregnancy test for female, demographics, medical history and physical examination. [a]Intervention means successful enrollment; [b]D means day from enrollment; [c]Life support therapy: vasopressor, invasive

mechanical ventilation and continuous renal replacement therapy; [d]Inflammatory mediators: interleukin -2(IL-2), IL-4, IL-6, IL-10, tumor necrosis factor-α (TNF-α), interferon-γ (IFN-γ), procalcitonin (PCT) and high-sensitivity C-reactive protein (hs-CRP); SOFA, Sequential Organ Failure Assessment score; APECHEⅡ, Acute Physiology and Chronic Health Evaluation; AKI, Acute Kidney Injury.
