# Supplementary material for: Adjuvant Lipoic acid Injection in Sepsis treatment in China (ALIS study): protocol for a randomised, single-blind, placebo-controlled trial
Source: BMJ Open. 2023 Jul 30;13(7):e072897. doi: 10.1136/bmjopen-2023-072897 (PMC10387639; doi:10.1136/bmjopen-2023-072897)
Supplement: Supplementary data [file bmjopen-2023-072897supp001.pdf]

知情同意书▪知情告知页

尊敬的先生/女士：

您好！您（或您的家属）当前所患的是□脓毒症□脓毒性休克。我们将邀请您（或您的家属）参加一项已上市药品治疗此疾病的临床研究：硫辛酸注射液治疗脓毒症及脓毒性休克成人患者的有效性及安全性的前瞻性、多中心、单盲、随机、安慰剂对照临床研究。该研究由茂名市人民医院、广东省人民医院发起。为了保证您的权益，本研究的研究方案和知情同意书均已获得伦理委员会的批准（编号：________________）。在同意参加这项研究之前，阅读并理解这份知情同意书对您来说十分重要。这份文件向您阐述了本研究的研究目的、步骤、可能给您带来的受益和风险等注意事项。请您仔细阅读，如有任何疑问请向负责该项研究的研究者提出。您参加本项研究是自愿的，并且可以在任何时间阶段退出研究。如果您决定参加这项研究，您将获得一份由双方签名的知情同意书副本。

一、研究背景与目的

脓毒症是机体对感染的反应失调而导致危及生命的器官功能障碍，脓毒性休克是指脓毒症合并出现严重的循环障碍和细胞代谢紊乱，是感染、烧/创伤、休克等急危重患者的严重并发症。脓毒症与脓毒性休克病死率高，大幅增加患者及家属经济负担。随着我国危重病监护救治技术的进步，脓毒症患者病死率虽然已显著下降，但仍高达 20~30%。针对脓毒症的病因治疗临床尚无特效药物。

研究表明，硫辛酸及其代谢产物是体内天然的生物抗氧化剂，鉴于氧化应激介导的细胞损伤是脓毒症重要病理生理学基础，通过捕捉体内自由基，从而抑制体内过度的炎症反应，改善脓毒症及脓毒性休克的治疗是可行的。我们拟在确诊脓毒症或脓毒性休克患者中应用硫辛酸治疗，探讨硫辛酸降低脓毒症及脓毒性休克患者病死率的有效性和安全性，以期降低患者病死率、提高救治成功率、缩短住院天数并改善改善预后。

二、研究内容和过程

本项研究采用前瞻性、多中心、单盲、随机、安慰剂对照临床研究设计，如果您符合本研究的入组标准，您将在脓毒症/脓毒性休克常规治疗（依据《拯救脓毒症和脓毒性休克运动指南》）或在常规治疗的基础上应用硫辛酸治疗，您分配在两种方案（硫辛酸组和安慰剂组）中接受治疗的概率是相同的。

本研究将记录您的个人情况和与疾病相关的临床资料：包括病史；常规医学检查（如血尿常规、肝肾功能、凝血功能、生化检查、血气分析、炎性因子和炎症标记物、影像学检查、

医学评分等）；为了客观评价病情变化，还将详细询问并记录您住院期间每天的病情变化，出院后随访至您入组后第 28 天。

以上治疗措施和医学检查项目，均为脓毒症和脓毒性休克诊断和治疗所必须的常规项目。本研究未设计超出常规诊断和治疗所必须的特殊检查和治疗项目，未增加患者的额外医疗费用。

三、参与本研究可能的受益

您和社会将可能从本研究中受益。此种受益包括您的病情有可能获得改善，减少器官损害的程度，硫辛酸免费用药，以及本研究可能帮助患有相似病情的其他患者。

无论您是否参加本研究，治疗与相关医学检查都会按本研究的常规方案进行。因此，参加本研究不会额外增加您的医疗负担；并且对您的病情观察和治疗将会更加全面、有利。

四、参与本研究可能的风险与不适

静脉滴注硫辛酸一般可能出现的不良反应有：例如头胀、呼吸困难、抽搐、复视、紫癜、出血倾向、过敏等。反应大多较轻且发生率较低，停药后可自行消失。

五、研究的保密性

如果您决定参加本项研究，您参加研究及在研究中的个人资料均属保密。您的血/尿等标本将以研究编号数字而非您的姓名加以标识。可以识别您身份的信息将不会透露给研究小组以外的成员，除非获得您的许可。所有的研究成员都被要求对您的身份保密。您的档案将保存在有锁的档案柜中，仅供研究人员查阅。为确保研究按照规定进行，必要时，政府管理部门或伦理委员会的成员按规定可以在研究单位查阅您的个人资料。这项研究结果发表时，将不会披露您个人的任何资料。

六、您的权利与义务

参加本研究期间需要您配合研究方案要求，接受研究医生对疾病预后的随访。

您可以选择不参加本项研究，或者在任何时候通知研究者要求退出研究，您做出的决定绝不影响您和医务人员的关系，您的任何医疗待遇、权益和医学关注不会因此受到影响，不会受到任何不公平的对待和惩罚。如果您决定退出研究，请您务必与您的研究医生联络，如果您在研究期间终止治疗和检查，本着对您的健康负责的目的，研究医生可能会询问一些与您健康相关的问题，还有可能要求您进行一些检查。

如果您需要其它治疗，或者没有遵循研究计划，或者您的主管研究医生认为您继续参与本研究不符合您的最大利益，研究医生可以让您退出研究；如果您在使用研究药物后出现不

适，或者安全风险，研究医生可能会在未征得您同意的情况下让您退出研究，研究医生将会与您讨论您退出研究后的相关事宜。

您可随时了解与本研究有关的信息资料和研究进展，如果您有与本研究有关的问题，或您在研究过程中发生了任何不适与损伤，或有关于本项研究参加者权益方面的问题您可以跟研究者联系。如果您对您作为研究参与者的权利有任何问题，您可以联系伦理委员会人员，电话：15915280880 。

致谢

医学科学的发展和进步离不开临床研究，您的参与将为医学科学进步做出贡献，对该疾病诊疗的研究与探索做出贡献。作为此项研究的研究者和申办者，我们将时刻铭记您的贡献，并对您表示最诚挚的感谢。

知情同意书▪同意签字页

在签署这份知情同意书前，我已经阅读上述信息，并且理解该项目的目的以及参加该项目可能带来的潜在利益和风险。我确认已经经过充分的考虑，也有机会对研究程序和研究药物提出疑问，并且所有的疑问均已得到令我满意的解答。

我同意研究医生收集和处理我的信息，包括与我健康有关的信息。我同意我的信息（个人信息除外）由研究单位处理。如果我决定退出本研究，我同意在此之前收集的信息仍可以被继续处理。

我有权在任何时候得到咨询服务，并有权决定在任何时候退出本研究计划而不会受到任何不利影响，不会因此丧失任何合法权利。我自愿签署这份知情同意书，并自愿参加此研究项目，会与研究者全面合作。我已得到此份文件的副本。

受试者

姓名(正楷) ____________________ 签名:____________________________

联系电话: _____________________ 日期: _____年____月____日

法定代理人/ 法定监护人

姓名(正楷) _________________ 签名:____________ 与受试者关系：____________

联系电话：_________________ 日期: _____年____月____日

公正的见证人声明

我确认，同意书中的信息被进行了准确的解释，已经被患者和/或患者的法定代理人理解，同意意见是由患者和/或患者的法定代理人自愿提供的。

姓名(正楷) ____________________ 签名:____________________________

联系电话: _____________________ 日期: _____年____月____日

注：如果患者、法定监护人或法定代理人不能阅读及签署同意书（例如重度视力受损、诵读困难或不识字），必须有至少 1 名公正的见证人。公正的见证人在讨论知情同意书的整个过程中都必须在场。

我已准确地将知情同意书内容告知受试者并对受试者的提问进行了解答，受试者自愿参加本项临床研究。

研究者签名：_____________ 联系电话：________________

日期: ____年____月____日
