# Supplementary material for: Adjuvant Lipoic acid Injection in Sepsis treatment in China (ALIS study): protocol for a randomised, single-blind, placebo-controlled trial
Source: BMJ Open. 2023 Jul 30;13(7):e072897. doi: 10.1136/bmjopen-2023-072897 (PMC10387639; doi:10.1136/bmjopen-2023-072897)
Supplement: Author's manuscript [file bmjopen-2023-072897.draft_revisions.pdf]

BMJ Open

BMJ Open is committed to open peer review. As part of this commitment we make the peer review history of every article we publish publicly available.

When an article is published we post the peer reviewers' comments and the authors' responses online. We also post the versions of the paper that were used during peer review. These are the versions that the peer review comments apply to.

The versions of the paper that follow are the versions that were submitted during the peer review process. They are not the versions of record or the final published versions. They should not be cited or distributed as the published version of this manuscript.

BMJ Open is an open access journal and the full, final, typeset and author-corrected version of record of the manuscript is available on our site with no access controls, subscription charges or pay-per-view fees (http://bmjopen.bmj.com).

If you have any questions on BMJ Open's open peer review process please email info.bmjopen@bmj.com

BMJ Open

Adjuvant Lipoic acid Injection in Sepsis treatment in China (ALIS study): study protocol for a randomized controlled trial

Journal:	*BMJ Open*
Manuscript ID	bmjopen-2023-072897
Article Type:	Protocol
Date Submitted by the Author:	20-Feb-2023

Complete List of Authors:	Hu, Linhui; Maoming People's Hospital, Department of Critical Care Medicine; Maoming People's Hospital, Clinical Research Center, Center of Scientific Research,<br>Zhou, Xinjuan; Maoming People's Hospital, Clinical Research Center, Center of Scientific Research; Maoming People's Hospital, Department of Cardiology<br>Huang, Jinbo; Maoming People's Hospital, Clinical Research Center, Center of Scientific Research<br>He, Yuemei; Maoming People's Hospital, Clinical Research Center, Center of Scientific Research; Maoming People's Hospital, Department of Cardiology<br>Wu, Quanzhong; Maoming People's Hospital, Department of Surgery Intensive Care Medicine<br>Huang, Xiangwei; Maoming People's Hospital, Department of Critical Care Medicine<br>Wu, Kunyong; Maoming People's Hospital, Center of Scientific Research<br>Wang, Guangwen; Maoming People's Hospital, Center of Scientific Research<br>Li, Sinian; Maoming People's Hospital, Department of Neurocritical Care Unit<br>Chen, Xiangyin; Maoming People's Hospital, Department of Surgery Intensive Care Medicine<br>Chen, Chunbo; Shenzhen People's Hospital, Department of Critical Care Medicine

Keywords:	Clinical trials < THERAPEUTICS, COMPLEMENTARY MEDICINE, Adult intensive & critical care < INTENSIVE & CRITICAL CARE

SCHOLARONE™ Manuscripts

Adjuvant Lipoic acid Injection in Sepsis treatment in China (ALIS study): study protocol for a randomized controlled trial

Linhui Hu[1,2, †], Xinjuan Zhou[2,3 †], Jinbo Huang[2, †], Yuemei He[2,3 †], Quanzhong Wu[4, †], Xiangwei Huang[1], Kunyong Wu[5], Guangwen Wang[5], Sinian Li[6], Xiangyin Chen[4], Chunbo Chen[7*]

[1]Department of Critical Care Medicine, Maoming People's Hospital, 101 Weimin Road, Maoming, 525000, Guangdong, China.

[2]Clinical Research Center, Center of Scientific Research, Maoming People's Hospital, 101 Weimin Road, Maoming, 525000, Guangdong, China.

[3]Department of Cardiology, Maoming People's Hospital, Maoming, China.

[4]Department of Surgery Intensive Care Medicine, Maoming People's Hospital, 101 Weimin Road, Maoming, 525000, Guangdong, China.

[5]Center of Scientific Research, Maoming People's Hospital, Maoming, 101 Weimin Road, Maoming, 525000, Guangdong, China.

[6]Department of Neurocritical Care Unit, Maoming People's Hospital, 101 Weimin Road, Maoming, 525000, Guangdong, China.

[7]Department of Critical Care Medicine, Shenzhen People's Hospital, The Second Clinical Medical College of Jinan University, The First Affiliated Hospital of Southern University of Science and Technology, No.1017 Dongmen North Road, Shenzhen 518000, Guangdong, China.

†These five authors contributed equally to this study as co-first authors.

*Corresponding author:

Chunbo Chen, gghccm@163.com

Department of Critical Care Medicine, Shenzhen People's Hospital, The Second Clinical Medical College of Jinan University, The First Affiliated Hospital of

For peer review only

Southern University of Science and Technology, No.1017 Dongmen North Road, Shenzhen 518000, Guangdong, China

Email addresses for all authors:

L.H.: hulinhui@live.cn

X.Z.: z1328314459@dingtalk.com

J.H.: 2589618856@qq.com

Y.H.: hym202008@163.com

Q.W.: wqz196@163.com

X.H.: 13828681930@163.com

K.W.: 237147873@qq.com

G.W.: 929233133@qq.com

S.L.: lisinian135455@163.com

X.C.: xychen569@163.com

C.C.: gghccm@163.com

For peer review only

ABSTRACT

Introduction Sepsis is a complex immune disorder that develops as a dysregulated host response to infection, which is a common but life-threatening condition with high mortality and morbidity in critically ill patients. Adjutant therapy is a beneficial complementary to sepsis treatment, and Lipoic acid has been widely demonstrated to attenuate sepsis-induced immune disorder in vivo and in vitro and protect organs from injury. However, evidence about the effect of clinically used lipoic acid injection on sepsis treatment is still lacking. Therefore, we designed this randomized controlled trial to explore lipoic acid injection's efficacy and safety in improving the prognosis of patients with sepsis or septic shock.

Methods and analysis A total of 352 patients with sepsis will be recruited into this trial. Eligible patients will undergo randomization at a 1:1 ratio to two arms: the control and experimental groups. The control group will receive routine treatment plus a placebo, while the experimental group will receive the lipoic acid injection. The primary efficacy endpoint of this study will be 28-day all-cause mortality. The secondary efficacy endpoints of this trial include many index pertaining to organ failure, mortality, stay length, mechanical ventilation duration and systematic inflammation status. All evaluations will be performed as planned at baseline and after enrolment. The researchers will assess clinical safety by analyzing adverse events and laboratory tests. All adverse events will be reported once recorded during the entire study procedure.

Ethics and dissemination Ethics Committee of Maoming People's Hospital approved this study (approval no. PJ2020MI-019-01). Study investigators will provide potential participants with verbal and written information before inclusion. Participants or their authorized representatives will provide informed consent. The results and conclusions of this clinical trial will be published at academic conferences or in journals.

Trial registration number ChiCTR2000039023.

Strengths and limitations of this study

This trial is a randomized controlled one with large sample size.

This trial eliminates the confounding factor of a terminal disease.

For peer review only

The hypothesis that lipoic acid protects organs from sepsis injury has adequate scientific evidence from animal and human research.

This trial is a single- rather than double-blind study design.

For peer review only

INTRODUCTION

Sepsis is a complex disorder associated with acute organ dysfunction that develops as a dysregulated host response to infection, and is the leading cause of death among patients in non-coronary intensive care units (ICUs) [1-3]. Despite the advances in sepsis management during the past decade, the mortality rate remains higher than 30%, up to 60% when septic shock presents [4]. In high-income countries, the population incidence rate for sepsis was 437 cases per 100,000 person-years, with 2.8 million deaths attributable to sepsis per year [5]. Du et al. [6] reported the standardized sepsis incidence and mortality of 461 and 79 cases per 100,000 person-years in China. The incidence will probably continue to increase with an aging Chinese population, which worsens the financial burden of treatment. Therefore, treating sepsis and septic shock is a patient safety priority.

Primary treatment of sepsis focus on timely intervention, including removal of the infection source, appropriate antibiotics, and restoration of tissue perfusion via fluid resuscitation and vasoactive drugs [7-9]. However, sepsis is a syndrome rather than a specific disease, making adjuvant therapies that intervene in its pathophysiological mechanism important beyond primary treatment. The 2021 guidelines only weakly recommend adjuvant therapies for managing sepsis [10], including using intravenous corticosteroids. Therefore, safe, effective, and inexpensive adjuvant treatments with less undesirable effects are badly required.

Although the precise mechanisms of sepsis-induced cell injury and organ dysfunction are not fully understood, it is well-recognized that simultaneous imbalanced activation of pro-inflammatory response (leading to cellular and tissue damage) and anti-inflammatory response (leading to immune system impairment) are associated [11-13]. Sepsis progression is linked with the imbalance between reactive oxygen species (ROS) and antioxidant enzymes. The lipoic acid (LA), or 1,2-dithiolane-3-pentanoic acid, is a naturally occurring dithiol compound synthesized enzymatically in the mitochondria from octanoic acid and exhibits unique antioxidant aspects. LA chelates several metal ions [14], scavenges ROS and reactive nitrogen species (RNS) in vitro and in vivo [15] to regenerate antioxidant molecules such as

For peer review only

glutathione, vitamin C , and vitamin E [16]. Remarkably, LA shows solubility both in water and lipids with its small chemical structure, which facilitates its access and permeability through the whole body to yield favorable effects.

Zhang et al. [17] show that LA effectively attenuates lipopolysaccharide (LPS)-induced acute inflammatory responses in vitro and in vivo by activating the phosphoinositide 3-kinase (PI3K)/protein kinase B (Akt) signaling pathway. Studies [18-21] demonstrate that LA reduces LPS-induced inflammation by inhibiting the activation of the nuclear factor κB (NF-κB) signaling pathway and suppressing excessive production levels of pro-inflammatory factors, which attenuates sepsis-induced inflammation and oxidative damage in organs by its anti-oxidative properties. LA significantly improves diabetic neurovascular and metabolic abnormalities [22] and may play a role in cardiovascular protection and as an anti-inflammatory agent [23-26]. Thus, we suggest that LA might be a potential adjuvant for sepsis treatment in protecting tissues from oxidative stress and preventing sepsis-induced organ dysfunction.

LA has been tested in different therapeutic situations, such as weight management [27 28], multiple sclerosis [29], male fertility [30] and type 2 diabetes [31 32], and promoted positive results. However, its preventive and therapeutic effects on sepsis-induced organ dysfunction are primarily investigated in rats [17 33 34]. To the best knowledge of us, as of today, there do not exist human studies on the therapeutic effects of LA in sepsis treatment. We, therefore, designed this prospective, multicenter, single-blinded, randomized control trial (RCT) to recruit patients in China who are diagnosed with sepsis or septic shock in ICU, to validate the efficacy and safety of LA injection over placebo in suppressing inflammation, attenuating organ dysfunction, shortening ICU or hospital stay, and improving patient outcomes including mortality.

For peer review only

METHODS AND ANALYSIS

Study Design

The Adjuvant Lipoic acid Injection in Sepsis treatment in China (ALIS study) is a prospective, multicenter, single-blind, randomized, placebo-controlled clinical trial in tertiary hospitals. This protocol and the template informed consent forms will be reviewed and approved by the applicable ethical committees (ECs) with respect to scientific content and compliance with applicable research and human subjects regulations in each participating centers. The departments involved in this study are general ICU, neurological ICU, surgical ICU, emergency ICU. Table 1 details the intended participating centers. The enrollment period is estimated to be two years, and the trial ended with the last follow-up. The research has been registered on chictr.org.cn (ChiCTR2000039023) [35]. The study design and protocol adhere to the Standard Protocol Items: Recommendations for Interventional Trials (SPIRIT) reporting guidelines. [36]

Protocol amendments

Protocol amendments will be documented with a brief description of the change and reference (date and number) when changes in the existing protocol significantly affect the safety of subjects, the scope of the investigation, or the scientific quality of the study. The coordinating investigator is obligated to notify this protocol amendments to all the investigators and the reviewing IRB and other relevant parties as appropriate.

For peer review only

Table 1 Research settings and names of each ethics committee

Research setting	Ethics committee name	Hospital rank and location
Maoming People's Hospital	Medical Ethics Committee of Maoming People's Hospital	Tertiary, Maoming, Guangdong, China
Guangdong Provincial People’s Hospital	Medical Ethics Committee of Guangdong Provincial People’s Hospital	Tertiary, Guangzhou, Guangdong, China
Affiliated Hospital of Guangdong medical university	Medical Ethics Committee of Affiliated Hospital of Guangdong medical university	Tertiary, Zhanjiang, Guangdong, China
Central People's Hospital of Zhanjiang	Medical Ethics Committee of Central People's Hospital of Zhanjiang	Tertiary, Zhanjiang, Guangdong, China
Huizhou Central Hospital	Medical Ethics Committee of Huizhou Central Hospital	Tertiary, Huizhou, Guangdong, China
Xiaolan People's Hospital of Zhongshan City	Medical Ethics Committee of Xiaolan People's Hospital of Zhongshan	Tertiary, Zhongshan, Guangdong, China
Third People's Hospital of Huizhou	Medical Ethics Committee of the Third People's Hospital of Huizhou	Tertiary, Huizhou, Guangdong, China
Yunfu People's Hospital	Medical Ethics Committee of Yunfu People's Hospital	Tertiary, Yunfu, Guangdong, China

For peer review only

Eligibility criteria

All the trial centers will use the same criteria for participant inclusion. The inclusion criteria are: (1) meeting the diagnostic criteria of Sepsis-3 for sepsis and septic shock formulated by the American Critical Care Medical Association/European Critical Care Medical Association [37]; (2) aged 18 - 80 years; (3) within 48 hours after sepsis or septic shock diagnosis; (4) having signed informed consent form.

The exclusion criteria are: (1) female patients in pregnancy or lactation; (2) presentation of persistent infection sources that cannot be eliminated by puncture, drainage, debridement, or other surgical operations; (3) patients who are taking part in another clinical trial; (4) patients being treated with other medications having antioxidant effects, such as vitamin C and vitamin E; (5) accompanied with non-infectious but life-threatening diseases, such as an advanced tumor, severe craniocerebral injury, uncontrollable massive bleeding, cardiogenic shock, etc.; (6) disapproval of comprehensive and active life support treatment; (7) patients who are allergic to LA or similar active agents (i.e., Vitamine B), and those who have previously been intolerant to the recommended dose of LA.

Interventions

The participants will be randomized to placebo or LA group and treated according to the corresponding treatment plan. Patients in the placebo group will receive a placebo consisting of 250 ml of normal saline, while in the treatment group patients will receive solution with LA injection of 600mg (Yabao Pharmaceutical Group Co., Ltd.) sloved in normal saline of 250 ml. Both the LA and the placebo were wrapped in aluminum platinum paper to avoid light, and the intravenous drip time must be less than 30 minutes. Concomitant care include routine use of a bundle of measures for the treatment of sepsis. The treating physicians are encouraged to adhere to the updated Surviving Sepsis Campaign guidelines [7], although the final decision is at the discretion of the treating physicians.

For peer review only

Evaluation and outcomes

Participants will be evaluated clinically and through laboratory testing according to supplementary table 1. The demographic data including age, gender, etc., disease diagnosis, progression, intervention and prognosis, and adverse events (AEs) will be recorded. Disease diagnosis data includes primary or concomitant diseases, blood culture results, white blood cell count, liver and kidney biochemistry, etc.. Disease progression data includes the baseline level of severity score and serum biomarkers, including sequential organ failure assessment (SOFA), Acute Physiology and Chronic Health Evaluation Ⅱ score, Procalcitonin, high-sensitivity C-reactive protein, Interleukin 2 (IL-2), IL-4, IL-6, IL-10, IL-α, Tumor necrosis factor (TNF)-α, Interferon-γ, as well as the follow-up value on Day 1 (D1), D3, D5, D7 after randomization, as well as the alteration value from baseline. Disease intervention data include the requirement and duration of life support including mechanical ventilation, vasoactive drugs, continuous renal replacement therapy, etc.. Disease prognosis data include 28-day all-cause mortality, ICU mortality, in-hospital mortality, incidence of new-onset acute kidney injury (AKI) after ICU admission, ICU stay, length of stay, etc.. We plan to collect data of organ dysfunction, the occurrence of AKI and safety events throughout the study.

The primary efficacy endpoint of this study is the 28-day all-cause mortality. The secondary efficacy endpoints include the following:

(1) Cumulative incidence of new-onset AKI after ICU admission to evaluate AKI incidence.

(2) ICU and in-hospital to evaluate mortality.

(3) Duration of mechanical ventilation, length of stay in ICU and hospital to evaluate resource cost.

(4) Alteration of SOFA score, use of vasopressors and CRRT to evaluate organ function.

For peer review only

(5) Alteration of lactic acid, procalcitonin, high-sensitivity C-reactive protein, IL-2, IL-4, IL-6, IL-10, TNF-α and Interferon-γ levels in the first week of enrollment to evaluate inflammatory status.

The safety endpoint includes any AEs, including serious adverse events (SAE), adverse drug reaction (head swelling, dyspnea, convulsion, diplopia, purpura, bleeding tendency, allergy, etc.).

Researchers should carefully observe any AEs of subjects during clinical research, and when AEs are found, researchers can take necessary measures according to their illness. Whether the AEs are related to LA injection or not, researchers should record them in detail in the case report form. The record of AEs should include: description of AEs and all related events, occurrence time, severity, duration, measures taken, final result and outcome, so as to analyze the correlation between AEs and LA injection, and the record should be signed and dated. If any SAE is encountered, it will be reported to the principal investigator of the trial, the ethics committee and the state supervision agency within 24 hours of its occurrence.When an AE is found, the researcher can take necessary treatment measures according to the illness, such as adjusting the dose, temporarily suspending the use of research drugs, etc., and decide whether to terminate the study. When SAE occurs, the researcher must immediately take necessary measures to protect the safety of the subjects.

For peer review only

Participant timeline

We assess the time to complete study enrolment to be 24 months. The follow-up frame is 28 days. At the time of the last visit of the last patient, data on the clinical status of all patients who have completed the trial or dropped out will be collected at the last visit of the last patient.

Sample size

This study took the 28-day all-cause mortality rate as the primary efficacy endpoint. Through a large-scale epidemiological survey in ICU, the 28-day all-cause mortality rate of the control group was 38.2%, and the LA injection treatment group's relative

risk reduction was about 0.33 in a RCT [38]. The 28-day all-cause mortality rate of the treatment group was expected to be 22.9%, with a one-sided test, $\alpha = 0.025$. $\beta = 0.2$ (power = 80%), threshold value $\Delta = 0$. Participants are randomized at a ratio of 1:1. The sample size for each group was calculated at 160 by a superiority test (lower is superior). Due to the drop-out inflation of about 10%, the sample size increased to 176 cases in each group.

Recruitment

To achieve adequate participant enrolment to reach target sample size, the trial is planned to conducted in several tertiary hospitals listed in Table 1. The enrollment period is estimated to be two years. A well-trained study coordinator in each participating center will be responsible for screening all potentially eligible patients based on the eligibility criteria.

Consent and assent

The clinical investigator ensures that informed consent is obtained from each research subject before that subject participates in the research study. When the research subject is disabled to consent for critical illness, informed consent (Appendix 1) is obtained from the authorized surrogates, for which the prior approval from the EC is a prerequisite. Note each EC will revise and adapt according to their own institution's guidelines. We will only obtain consent to use data and samples for the research question described in this protocol. Thus, we do not intend to use participant data or biological samples in ancillary studies.

Allocation

Participants with a diagnosis of sepsis, according to the Sepsis-3 criteria, enter the screening. Participants who meet the eligibility criteria will be randomly assigned to the treatment and control group with a 1:1 allocation as per a computer-generated block (size=8) randomization schedule stratified by site. Four patients in each block received the LA injection, and the other four received a placebo. The randomization

For peer review only

method and block size will not be blinded until all data analysis is completed. The investigators did not participate in data collection. The randomization center randomly assigned the screened patients at each hospital through net verification. Researchers are blind to patient allocation orders. To maintain the overall quality and legitimacy of the clinical trial, code breaks should occur only in exceptional circumstances when knowledge of the actual treatment is absolutely essential for further management of the patient. Investigators are encouraged to discuss with the medical advisors if he/she believes that unblinding is necessary, and must report all code breaks (with reason) as they occur on the corresponding CRF page. Unblinding should not necessarily be a reason for study drug discontinuation.

Data collection and management

Data collectors, researchers, coordinators, etc., will have received good training in advance to be competent in managing the project. Coordinators of each center use the electronic data acquisition system (https://www.mmphcrc.com/pdf/medicalHistory/lxs crf.html) for case report form input. This system obtained the computer software's copyright on April 6, 2022 (Software No. 9387742). After data entry, a particular person shall regularly check the accuracy of data entry. When it is necessary to revise the data, the modified data shall be reviewed by the data manager. Data access will be strictly restricted. The principal investigators of each center can log in to the system to view the data and only get full access to the information with the permission of the coordinating investigators of this research. All research documents will be. All study-related information will be treated as highly confidential and stored securely at the study site.

Data monitoring

An independent Data and Safety Monitoring Board will be responsible for data monitoring and blinded analysis. Committee members regularly review the research data. The trial is also examined by the medical ethics committee of each participating center.

For peer review only

Analyses set

Analysis will be done considering the “intention to treat” principle. Patients who complete the randomization, regardless of whether they completed the trial or received the treatment in the designated group, are retained in the original group for analysis. The randomization information is maintained to the maximum extent.

Intention-to-treat set (ITTS)

ITTS refers to the ideal population of subjects meeting the ITT principles. To best retain the randomized information, ensure that the differences in trial results are attributed to the differences in treatment, and make the effect of treatment (postpyloric feeding) best assessed, the principle of ITT analysis is adopted. ITT set (ITTS) included all randomized patients, regardless of whether they have received the treatment of the allocated group, completed the trial, all of whom remain in the original group for analysis.

Per protocol set (PPS)

PPS refers to all participants who fulfill the eligibility criteria and receive treatment per randomization. They have good compliance with the trial protocol, such as being ready to receive treatment and measurement for the primary efficacy endpoints, with the required contents in the CRF filled. PPS is used to analyze the primary efficacy endpoints.

Security set (SS)

The SS included all actual cases that have received at least one administration of assigned fluid per randomization, with the safety endpoints recorded. The incidence of adverse events is denominated by the number of patients in the safety set.

Statistical analysis

All analyses will be performed according to the intent-to treat (ITT) principle. The primary analyses will be performed according to the intention-to-treat principle. The

For peer review only

data are statistically processed by with the software R v3.3.3 (R Foundation for Statistical Computing, Vienna, Austria) using RStudio v1.0.136 (RStudio Inc, Boston, MA, USA). Data will be described according to their distribution. Continuous data are indicated with mean ± standard deviation or median and interquartile range in agreement with the data while categorical data are reported with number and percentage. The comparison between groups are performed by group t-test or variance analysis. If normality is not assumed, non-parametric tests will be used. Categorical results are compared by χ^2 test or Fisher's Exact test. The statistical significance level for all the tests is set at a p value < 0.05.

Interim analysis

No interim analysis is planned for this trial.

Patient and public involvement

Patients or the public were not involved in the design, or conduct, or reporting, or dissemination plans of our research.

Ancillary and post-trial care

All patients would be treated, monitored, and routinely assessed regarding the development of Sepsis campaign and recovery process of Sepsis in each participating study center in China.

ETHICS AND DISSEMINATION

Ethics approval

This study was approved by the Ethics Committee of Maoming People's Hospital, China. Approval (NO. PJ2020MI-019-01) was released on September 25, 2020. Before being included in the study, all participants will get the informed consent approved by the ethics committee. After entering the study, it will be carried out in strict accordance with the clinical research plan and according to the Declaration of Helsinki.

For peer review only

Informed consent forms and privacy protection

In this study, subjects are not allowed to join the group until they have obtained a signed written informed consent form. During the participants' participation, all updated versions of informed consent forms and written information will be provided to the participants. Informed consent forms should be kept as important documents of clinical research for future reference. If there is any change in the informed consent form in this study, the revised informed consent form can only be used after the written approval of the ethics committee, and participants must re-sign the revised version.

Participants in the project confirm and support the right guidelines for protecting subjects' privacy from infringement. During the whole process of this study, the original data of the subjects will only be connected with the clinical research database or files of the participating units of the project through a unique identification number. With the approval of applicable laws and regulations, limited subject characteristics such as gender, age or date of birth and initials of the subject's name can be used to verify the accuracy of the subject and the subject's unique identification number.

Dissemination

Results will be presented at relevant national and international conferences and published in peer-reviewed journals.

DISCUSSION

Sepsis is defined as life-threatening organ dysfunction resulting from dysregulated host responses to infection and codified by an acute change in SOFA score of 2 points or more [39]. As a subset of sepsis, septic shock is a condition in which underlying circulatory, cellular, and metabolic abnormalities are profound enough to develop hypotension requiring vasopressors to maintain a mean arterial blood pressure above 65 mmHg with an elevated serum lactate concentration of more than 2 mmol/L, despite adequate fluid resuscitation.

For peer review only

As a global health priority in 2017 made by the World Health Assembly [40], sepsis and septic shock is a common condition associated with unacceptably high mortality and, for many of those who survive, long-term morbidity. Most data are from high-income countries, where 2.8 million deaths yearly are attributable to sepsis. Du et al. [6] reported the standardized sepsis incidence and mortality of 461 and 79 cases per 100,000 person-years, respectively, in China. It is conceivable that the data may be higher due to relatively lower awareness of sepsis and accessibility of organ support from intensive care. Although sepsis is a global priority, most available papers and evidence come disproportionately from high-income countries. More research on the pharmacological agent that can improve outcomes by intervening with the disease's pathophysiological mechanism is especially needed in low-income and middle-income countries like China, which hold a large population of over 1.4 billion.

As we know, sepsis is characterized by a systemic dysregulated host response to infection. One well-described pathway of immune activation in response to infection occurs when highly conserved microbial pathogen-associated molecular patterns are recognized by pattern-recognition receptors, including Toll-like receptors, on the innate immune system cells. This interaction triggers the release of pro-inflammatory and anti-inflammatory mediators by activating NF-κB and neutrophils. Cytokines, such as TNF-α, IL-1, IL-2, IL-6, IL-8, and others, cause neutrophil-endothelial cell adhesion, activate the complement and clotting cascades, also known as "inflammatory storm".

The PI3K/Akt pathway, known as a conserved family of signal transduction enzymes, is involved in regulating cellular proliferation and survival. There are substantial differences among different cell types regarding the anti-inflammatory role of the PI3K/Akt pathway, which either acts as a positive [41-43] or negative regulator [44-46] of NF-κB activation and cytokine production, depending on the nature of the stimulus and the cell type. A growing body of evidence suggests that the PI3K/Akt pathway plays an essential role as a negative feedback regulator of excessive innate immune and Toll-like receptor-mediated pro-inflammatory responses [44-51]. Many studies [44 47-49 52 53] indicate that the PI3K/Akt pathway limits inflammation in

endotoxemia and sepsis, whereas inhibition of PI3K/Akt signaling enhanced LPS-induced activation of NF-κB and AP-1 and gene expression of TNF-α and tissue factor in cultured cells [44]. It is well documented that NF-κB plays a prominent role in LPS-induced transcriptional regulation of most inflammatory genes that contribute to the development of septic shock, multiple organ failure, and death [54-57]. Of clinical relevance, NF-κB binding activity was increased in patients with acute inflammation and sepsis and correlated with clinical severity and mortality [58 59]. Therefore, LA may well suppress septic inflammation through its role in PI3K/Akt pathway.

On the other side, oxidative stress, a major promoter, and mediator of the systemic inflammatory response, is considered pivotal in the pathophysiology of sepsis [60]. The early phase of sepsis is characterized by a massive formation of ROS and RNS, such as superoxide and nitric oxide. Excessive production of ROS and RNS during inflammation is associated with the occurrence of sepsis, and a massive increase in radical species can lead to marked oxidative stress, promoting cell injury and death, resulting in a breakdown of antioxidant systems, and initiating mitochondrial dysfunction, which may result in organ damage [61 62]. Normally, a complex system of interacting antioxidant defenses can combat oxidative stress and prevent damage to mitochondria. In sepsis, a state of severe oxidative stress is encountered, with host endogenous antioxidant defenses overcome and the redox (reduction-oxidation) cellular balance broken down. It has been suggested that antioxidant therapy delivered specifically to mitochondria, which augments the endogenous antioxidant defenses, has the potential to protect against such radical-induced injury [63 64]. Thus, LA is probably useful in preventing organs from injury as a suppressor of ROS.

Over in all, LA, one agent modulating the inflammatory cascades through the above-mentioned mechanisms, is potentially effective in modulating the immune imbalance and decreasing ensued organ impairment in septic patients. Actually, LA has been demonstrated to be favorable in different therapeutic situations, such as weight management [27 28], multiple sclerosis [29], male fertility [30], and type 2 diabetes [31 32]. Therefore, we design this study protocol for a randomized controlled trial to

For peer review only - http://bmjopen.bmj.com/site/about/guidelines.xhtml

explore the efficacy and safety of LA injection in sepsis treatment in China (ALIS study).

There are some strengths of this study design. First, this RCT with a large sample size can help us to increase our understanding of the value of LA, a commonly used but non-evidence-based adjuvant drug in sepsis treatment. Second, we eliminate the confounding factor of a terminal disease. Sepsis can be a terminal event in patients already dying from other causes (e.g., terminal cancer). Baseline mortality from sepsis is likely part of the nature of the syndrome itself. Third, the hypothesis that LA protects organs from sepsis injury is based on adequate evidence from animal and human research. However, there remain some limitations that cannot be avoided. First, the number of participating centers is relatively less, which may lead to selective bias impacting the transferability of the research results to other contexts or settings. Second, this is a single-blind study design, which may lead to the investigator's diagnostic bias, causing a treatment imbalance between the treatment and control group.

In summary, the ALIS study is a large-sample-sized, multicenter, single-blinded, randomized, parallel-group, controlled trial of PTF in China. It is well designed based on robust evidence from base and clinical studies. This trial's results may help provide evidence-based recommendations for the prevention of sepsis in critically ill patients.

Acknowledgments

Not applicable.

Contributors

CBC conceived of the study. LHH, XJZ, JBH, YMH, and QZW initiated the study design and XWH, KYW, GWW, SNL and XYC helped with implementation. CBC and LHH are grant holders. LHH, YMH and XJZ provided statistical expertise in clinical trial design. LHH and XJZ drafted the manuscript. CBC helped draft and revise the manuscript. All authors contributed to refinement of the study protocol and approved the final manuscript.

For peer review only

Funding

Author Chunbo Chen is currently receiving a grant (#MaoRenCaiBan [2020]24) from the Office of Talent Work Leading Team in Maoming, a grant (#201803010058) from the Guangzhou Science and Technology Program, a grant (#82172162) from the National Natural Science Foundation of China, and a grant (#DFJH2020028) under the Major Program of Summit Project, Guangdong Province High-level Hospital Construction Project of Guangdong Provincial People's Hospital, Guangdong Academy of Medical Sciences. Author Linhui Hu is currently receiving a grant (#2020YJ01) from the Emergent Science and Technology Project for Prevention and Treatment of Novel Coronavirus Pneumonia, a grant (#zx2020017) from the High-level Hospital Construction Research Project of Maoming People's Hospital, and a grant (#SY2021005) from Excellent Young Talents Project of Maoming People's Hospital, a grant (#B2022246) from Medical Research Fund of Guangdong Province, and a grant (#2021KJZXZJYX003) from Special Science and Technology Fund of Maoming City. The study was supported by the Special Science and Technology Fund of Maoming City.

Disclaimer

The funders had no role in the design and conduct of the study; collection, management, analysis, and interpretation of the data; preparation, writing, review, or approval of the report; and decision to submit the report for publication.

Competing interests

All the other authors declare that they have no competing interests.

Patient consent for publication

Not applicable.

Provenance and peer review

Not commissioned; externally peer reviewed.

For peer review only

References

1. Angus DC, Linde-Zwirble WT, Lidicker J, et al. Epidemiology of severe sepsis in the United States: analysis of incidence, outcome, and associated costs of care. *Crit Care Med* 2001;29(7):1303-10. doi: 10.1097/00003246-200107000-00002 [published Online First: 2001/07/11]
2. Hu L, Gao L, Zhang D, et al. The incidence, risk factors and outcomes of acute kidney injury in critically ill patients undergoing emergency surgery: a prospective observational study. *BMC Nephrol* 2022;23(1):42. doi: 10.1186/s12882-022-02675-0 [published Online First: 2022/01/24]
3. Chao J, Cui S, Liu C, et al. Detection of Early Cytokine Storm in Patients with Septic Shock After Abdominal Surgery. *J Transl Int Med* 2020;8(2):91-98. doi: 10.2478/jtim-2020-0014 [published Online First: 2020/09/29]
4. Cohen J, Vincent JL, Adhikari NK, et al. Sepsis: a roadmap for future research. *Lancet Infect Dis* 2015;15(5):581-614. doi: 10.1016/s1473-3099(15)70112-x [published Online First: 2015/05/02]
5. Adhikari NK, Fowler RA, Bhagwanjee S, et al. Critical care and the global burden of critical illness in adults. *Lancet* 2010;376(9749):1339-46.
6. Jiang W, Yu X, Sun T, et al. ADJunctive Ulinastatin in Sepsis Treatment in China (ADJUST study): study protocol for a randomized controlled trial. *Trials* 2018;19(1):133.
7. Dellinger RP, Levy MM, Rhodes A, et al. Surviving Sepsis Campaign: international guidelines for management of severe sepsis and septic shock, 2012. *Intensive Care Med* 2013;39(2):165-228. doi: 10.1007/s00134-012-2769-8 [published Online First: 2013/01/31]
8. Dellinger RP, Levy MM, Carlet JM, et al. Surviving Sepsis Campaign: international guidelines for management of severe sepsis and septic shock: 2008. *Crit Care Med* 2008;36(1):296-327. doi: 10.1097/01.Ccm.0000298158.12101.41 [published Online First: 2007/12/26]
9. Hollenberg SM, Ahrens TS, Annane D, et al. Practice parameters for hemodynamic support of sepsis in adult patients: 2004 update. *Crit Care Med* 2004;32(9):1928-48. doi: 10.1097/01.ccm.0000139761.05492.d6 [published Online First: 2004/09/03]
10. Rehn M, Chew MS, Olkkola KT, et al. Surviving sepsis campaign: International guidelines for management of sepsis and septic shock in adults 2021 - endorsement by the Scandinavian society of anaesthesiology and intensive care medicine. *Acta Anaesthesiol Scand* 2022;66(5):634-35.
11. Hotchkiss RS, Karl IE. The pathophysiology and treatment of sepsis. *N Engl J Med* 2003;348(2):138-50. doi: 10.1056/NEJMra021333 [published Online First: 2003/01/10]
12. Hotchkiss RS, Monneret G, Payen D. Sepsis-induced immunosuppression: from cellular dysfunctions to immunotherapy. *Nat Rev Immunol* 2013;13(12):862-74.
13. Hotchkiss RS, Opal S. Immunotherapy for sepsis--a new approach against an ancient foe. *N Engl J Med* 2010;363(1):87-9.

For peer review only

14. Ou P, Tritschler HJ, Wolff SP. Thioctic (lipoic) acid: a therapeutic metal-chelating antioxidant? *Biochem Pharmacol* 1995;50(1):123-6. doi: 10.1016/0006-2952(95)00116-h [published Online First: 1995/06/29]
15. Rochette L, Ghibu S, Richard C, et al. Direct and indirect antioxidant properties of α-lipoic acid and therapeutic potential. *Molecular Nutrition & Food Research* 2013;57(1):114-25. doi: https://doi.org/10.1002/mnfr.201200608
16. Shay KP, Moreau RF, Smith EJ, et al. Alpha-lipoic acid as a dietary supplement: molecular mechanisms and therapeutic potential. *Biochim Biophys Acta* 2009;1790(10):1149-60.
17. Zhang WJ, Wei H, Hagen T, et al. Alpha-lipoic acid attenuates LPS-induced inflammatory responses by activating the phosphoinositide 3-kinase/Akt signaling pathway. *Proc Natl Acad Sci U S A* 2007;104(10):4077-82.
18. Demarco VG, Scumpia PO, Bosanquet JP, et al. alpha-lipoic acid inhibits endotoxin-stimulated expression of iNOS and nitric oxide independent of the heat shock response in RAW 264.7 cells. *Free Radic Res* 2004;38(7):675-82. doi: 10.1080/10715760410001702503 [published Online First: 2004/09/30]
19. Della Giustina A, Goldim MP, Danielski LG, et al. Alpha-lipoic acid attenuates acute neuroinflammation and long-term cognitive impairment after polymicrobial sepsis. *Neurochem Int* 2017;108:436-47. doi: 10.1016/j.neuint.2017.06.003 [published Online First: 2017/06/14]
20. Petronilho F, Florentino D, Danielski LG, et al. Alpha-Lipoic Acid Attenuates Oxidative Damage in Organs After Sepsis. *Inflammation* 2016;39(1):357-65. doi: 10.1007/s10753-015-0256-4 [published Online First: 2015/10/04]
21. Li G, Fu J, Zhao Y, et al. Alpha-lipoic acid exerts anti-inflammatory effects on lipopolysaccharide-stimulated rat mesangial cells via inhibition of nuclear factor kappa B (NF-κB) signaling pathway. *Inflammation* 2015;38(2):510-9. doi: 10.1007/s10753-014-9957-3 [published Online First: 2014/06/26]
22. Ziegler D. Thioctic acid for patients with symptomatic diabetic polyneuropathy: a critical review. *Treat Endocrinol* 2004;3(3):173-89. doi: 10.2165/00024677-200403030-00005 [published Online First: 2005/07/20]
23. Melhem A, Stern M, Shibolet O, et al. Treatment of chronic hepatitis C virus infection via antioxidants: results of a phase I clinical trial. *J Clin Gastroenterol* 2005;39(8):737-42. doi: 10.1097/01.mcg.0000174023.73472.29 [published Online First: 2005/08/06]
24. Sola S, Mir MQ, Cheema FA, et al. Irbesartan and lipoic acid improve endothelial function and reduce markers of inflammation in the metabolic syndrome: results of the Irbesartan and Lipoic Acid in Endothelial Dysfunction (ISLAND) study. *Circulation* 2005;111(3):343-8. doi: 10.1161/01.Cir.0000153272.48711.B9 [published Online First: 2005/01/19]
25. Cho YS, Lee J, Lee TH, et al. alpha-Lipoic acid inhibits airway inflammation and hyperresponsiveness in a mouse model of asthma. *J Allergy Clin Immunol* 2004;114(2):429-35. doi: 10.1016/j.jaci.2004.04.004 [published Online First: 2004/08/19]

For peer review only

26. Jesudason EP, Masilamoni JG, Jesudoss KS, et al. The protective role of DL-alpha-lipoic acid in the oxidative vulnerability triggered by Abeta-amyloid vaccination in mice. *Mol Cell Biochem* 2005;270(1-2):29-37. doi: 10.1007/s11010-005-3301-z [published Online First: 2005/03/29]
27. Kim NW, Song YM, Kim E, et al. Adjunctive α-lipoic acid reduces weight gain compared with placebo at 12 weeks in schizophrenic patients treated with atypical antipsychotics: a double-blind randomized placebo-controlled study. *Int Clin Psychopharmacol* 2016;31(5):265-74. doi: 10.1097/yic.0000000000000132 [published Online First: 2016/06/09]
28. Li N, Yan W, Hu X, et al. Effects of oral α-lipoic acid administration on body weight in overweight or obese subjects: a crossover randomized, double-blind, placebo-controlled trial. *Clin Endocrinol (Oxf)* 2017;86(5):680-87. doi: 10.1111/cen.13303 [published Online First: 2017/02/28]
29. Khalili M, Eghtesadi S, Mirshafiey A, et al. Effect of lipoic acid consumption on oxidative stress among multiple sclerosis patients: a randomized controlled clinical trial. *Nutr Neurosci* 2014;17(1):16-20. doi: 10.1179/1476830513y.0000000060 [published Online First: 2013/03/15]
30. Haghighian HK, Haidari F, Mohammadi-Asl J, et al. Randomized, triple-blind, placebo-controlled clinical trial examining the effects of alpha-lipoic acid supplement on the spermatogram and seminal oxidative stress in infertile men. *Fertil Steril* 2015;104(2):318-24. doi: 10.1016/j.fertnstert.2015.05.014 [published Online First: 2015/06/09]
31. Ametov AS, Barinov A, Dyck PJ, et al. The sensory symptoms of diabetic polyneuropathy are improved with alpha-lipoic acid: the SYDNEY trial. *Diabetes Care* 2003;26(3):770-6. doi: 10.2337/diacare.26.3.770 [published Online First: 2003/03/01]
32. Garcia-Alcala H, Santos Vichido CI, Islas Macedo S, et al. Treatment with α-Lipoic Acid over 16 Weeks in Type 2 Diabetic Patients with Symptomatic Polyneuropathy Who Responded to Initial 4-Week High-Dose Loading. *J Diabetes Res* 2015;2015:189857.
33. Shen HH, Lam KK, Cheng PY, et al. Alpha-lipoic acid prevents endotoxic shock and multiple organ dysfunction syndrome induced by endotoxemia in rats. *Shock* 2015;43(4):405-11. doi: 10.1097/shk.0000000000000295 [published Online First: 2014/12/17]
34. Cadirci E, Altunkaynak BZ, Halici Z, et al. Alpha-lipoic acid as a potential target for the treatment of lung injury caused by cecal ligation and puncture-induced sepsis model in rats. *Shock* 2010;33(5):479-84. doi: 10.1097/SHK.0b013e3181c3cf0e [published Online First: 2009/10/14]
35. Chictr.org.cn. Lipoic acid injection in the treatment of adult patients with sepsis and septic shock: a prospective, multicenter, single blind, randomized, placebo-controlled clinical trial 2021 [Available from: https://www.chictr.org.cn/showproj.aspx?proj=62629 accessed 02/14 2023.

For peer review only

36. Chan AW, Tetzlaff JM, Gotzsche PC, et al. SPIRIT 2013 explanation and elaboration: guidance for protocols of clinical trials. *BMJ* 2013;346:e7586. doi: 10.1136/bmj.e7586 [published Online First: 2013/01/11]
37. Shankar-Hari M, Phillips GS, Levy ML, et al. Developing a New Definition and Assessing New Clinical Criteria for Septic Shock: For the Third International Consensus Definitions for Sepsis and Septic Shock (Sepsis-3). *JAMA* 2016;315(8):775-87. doi: 10.1001/jama.2016.0289 [published Online First: 2016/02/24]
38. Hejazi N, Mazloom Z, Zand F, et al. The Beneficial Effects of α-Lipoic Acid in Critically Ill Patients: A Prospective, Randomized, Double-Blind, Placebo-Controlled Trial. *Asian Journal of Anesthesiology* 2018;56(2):45-55.
39. Qu R, Hu L, Ling Y, et al. C-reactive protein concentration as a risk predictor of mortality in intensive care unit: a multicenter, prospective, observational study. *BMC Anesthesiol* 2020;20(1):292. doi: 10.1186/s12871-020-01207-3 [published Online First: 2020/11/24]
40. Mghamba JM, Talisuna AO, Suryantoro L, et al. Developing a multisectoral National Action Plan for Health Security (NAPHS) to implement the International Health Regulations (IHR 2005) in Tanzania. *BMJ Glob Health* 2018;3(2):e000600.
41. Ozes ON, Mayo LD, Gustin JA, et al. NF-kappaB activation by tumour necrosis factor requires the Akt serine-threonine kinase. *Nature* 1999;401(6748):82-5. doi: 10.1038/43466 [published Online First: 1999/09/15]
42. Thomas KW, Monick MM, Staber JM, et al. Respiratory syncytial virus inhibits apoptosis and induces NF-kappa B activity through a phosphatidylinositol 3-kinase-dependent pathway. *J Biol Chem* 2002;277(1):492-501. doi: 10.1074/jbc.M108107200 [published Online First: 2001/11/01]
43. Madrid LV, Mayo MW, Reuther JY, et al. Akt stimulates the transactivation potential of the RelA/p65 Subunit of NF-kappa B through utilization of the Ikappa B kinase and activation of the mitogen-activated protein kinase p38. *J Biol Chem* 2001;276(22):18934-40. doi: 10.1074/jbc.M101103200 [published Online First: 2001/03/22]
44. Guha M, Mackman N. The phosphatidylinositol 3-kinase-Akt pathway limits lipopolysaccharide activation of signaling pathways and expression of inflammatory mediators in human monocytic cells. *J Biol Chem* 2002;277(35):32124-32. doi: 10.1074/jbc.M203298200 [published Online First: 2002/06/08]
45. Kim I, Oh JL, Ryu YS, et al. Angiopoietin-1 negatively regulates expression and activity of tissue factor in endothelial cells. *FASEB J* 2002;16(1):126-8. doi: 10.1096/fj.01-0556fje [published Online First: 2001/12/01]
46. Bommhardt U, Chang KC, Swanson PE, et al. Akt decreases lymphocyte apoptosis and improves survival in sepsis. *J Immunol* 2004;172(12):7583-91. doi: 10.4049/jimmunol.172.12.7583 [published Online First: 2004/06/10]
47. Schabbauer G, Tencati M, Pedersen B, et al. PI3K-Akt pathway suppresses coagulation and inflammation in endotoxemic mice. *Arterioscler Thromb Vasc*

Biol 2004;24(10):1963-9. doi: 10.1161/01.ATV.0000143096.15099.ce [published Online First: 2004/08/21]

48. Williams DL, Li C, Ha T, et al. Modulation of the phosphoinositide 3-kinase pathway alters innate resistance to polymicrobial sepsis. *J Immunol* 2004;172(1):449-56. doi: 10.4049/jimmunol.172.1.449 [published Online First: 2003/12/23]
49. Rajaram MV, Ganesan LP, Parsa KV, et al. Akt/Protein kinase B modulates macrophage inflammatory response to Francisella infection and confers a survival advantage in mice. *J Immunol* 2006;177(9):6317-24. doi: 10.4049/jimmunol.177.9.6317 [published Online First: 2006/10/24]
50. Fukao T, Koyasu S. PI3K and negative regulation of TLR signaling. *Trends Immunol* 2003;24(7):358-63. doi: 10.1016/s1471-4906(03)00139-x [published Online First: 2003/07/16]
51. Martin M, Rehani K, Jope RS, et al. Toll-like receptor-mediated cytokine production is differentially regulated by glycogen synthase kinase 3. *Nat Immunol* 2005;6(8):777-84.
52. Matsuda N, Hayashi Y, Takahashi Y, et al. Phosphorylation of endothelial nitric-oxide synthase is diminished in mesenteric arteries from septic rabbits depending on the altered phosphatidylinositol 3-kinase/Akt pathway: reversal effect of fluvastatin therapy. *J Pharmacol Exp Ther* 2006;319(3):1348-54. doi: 10.1124/jpet.106.109785 [published Online First: 2006/09/28]
53. Ong E, Gao XP, Predescu D, et al. Role of phosphatidylinositol 3-kinase-gamma in mediating lung neutrophil sequestration and vascular injury induced by E. coli sepsis. *Am J Physiol Lung Cell Mol Physiol* 2005;289(6):L1094-103. doi: 10.1152/ajplung.00179.2005 [published Online First: 2005/09/27]
54. Collins T, Read MA, Neish AS, et al. Transcriptional regulation of endothelial cell adhesion molecules: NF-kappa B and cytokine-inducible enhancers. *FASEB J* 1995;9(10):899-909. [published Online First: 1995/07/01]
55. Karin M, Liu Z, Zandi E. AP-1 function and regulation. *Curr Opin Cell Biol* 1997;9(2):240-6. doi: 10.1016/s0955-0674(97)80068-3 [published Online First: 1997/04/01]
56. Liu H, Colavitti R, Rovira, II, et al. Redox-dependent transcriptional regulation. *Circ Res* 2005;97(10):967-74. doi: 10.1161/01.Res.0000188210.72062.10 [published Online First: 2005/11/15]
57. Liu SF, Malik AB. NF-kappa B activation as a pathological mechanism of septic shock and inflammation. *Am J Physiol Lung Cell Mol Physiol* 2006;290(4):L622-L45. doi: 10.1152/ajplung.00477.2005 [published Online First: 2006/03/15]
58. Böhrer H, Qiu F, Zimmermann T, et al. Role of NFkappaB in the mortality of sepsis. *J Clin Invest* 1997;100(5):972-85.
59. Arnalich F, Garcia-Palomero E, López J, et al. Predictive value of nuclear factor kappaB activity and plasma cytokine levels in patients with sepsis. *Infect Immun* 2000;68(4):1942-5.

For peer review only

60. Miliaraki M, Briassoulis P, Ilia S, et al. Oxidant/Antioxidant Status Is Impaired in Sepsis and Is Related to Anti-Apoptotic, Inflammatory, and Innate Immunity Alterations. *Antioxidants (Basel)* 2022;11(2)
61. Crimi E, Sica V, Slutsky AS, et al. Role of oxidative stress in experimental sepsis and multisystem organ dysfunction. *Free Radic Res* 2006;40(7):665-72. doi: 10.1080/10715760600669612 [published Online First: 2006/09/21]
62. Joffre J, Hellman J. Oxidative Stress and Endothelial Dysfunction in Sepsis and Acute Inflammation. *Antioxid Redox Signal* 2021;35(15):1291-307. doi: 10.1089/ars.2021.0027 [published Online First: 2021/02/28]
63. Galley HF. Oxidative stress and mitochondrial dysfunction in sepsis. *Br J Anaesth* 2011;107(1):57-64. doi: 10.1093/bja/aer093 [published Online First: 2011/05/21]
64. Goode HF, Webster NR. Free radicals and antioxidants in sepsis. *Crit Care Med* 1993;21(11):1770-6. doi: 10.1097/00003246-199311000-00029 [published Online First: 1993/11/01]

For peer review only

Supplementary Table 1 Trial schedule of the LAS study

Timepoint	Baseline	[a]Interventions									Termination	Follow-up period
	[b]D0	D1	D2	D3	D4	D5	D6	D7	D8-10	D…	D28	D…
Eligibility screening	×											
Informed consent obtaining	×											
Randomization	×											
Medical history collection	×											
Population data collection	×											
Baseline data collection	×											
Formulation of drug administration plan	×											
Evaluation of drug-related adverse reactions		×	×	×	×	×	×	×	×			
Evaluate serious adverse events		×	×	×	×	×	×	×	×			

For peer review only

Timepoint	Baseline	[a]Interventions									Termination	Follow-up period
	[b]D0	D1	D2	D3	D4	D5	D6	D7	D8-10	D…	D28	D…
Vital signs	×	×	×	×	×	×	×	×			×	
SOFA/APACHEIIscore	×	×		×		×		×				
[c]Need and duration of life support treatment		×	×	×	×	×	×	×	×	×	×	
[d]Levels of inflammatory mediators	×	×		×		×		×				
Arterial lactic acid level	×	×		×		×		×				
Transfer out of ICU		×	×	×	×	×	×	×	×	×	×	×
ICU death time		×	×	×	×	×	×	×	×	×	×	×
AKI occurrence time		×	×	×	×	×	×	×	×	×	×	×
Hospital death time		×	×	×	×	×	×	×	×	×	×	×
Discharge time		×	×	×	×	×	×	×	×	×	×	×

Eligibility screening includes diagnosis, inclusion/exclusion criteria, urine pregnancy test for female, demographics, medical history and physical examination. [a]Intervention means successful enrollment; [b]D means day from enrollment; [c]Life support therapy: vasopressor, invasive

For peer review only

mechanical ventilation and continuous renal replacement therapy; [d]Inflammatory mediators: interleukin -2(IL-2), IL-4, IL-6, IL-10, tumor necrosis factor-α (TNF-α), interferon-γ (IFN-γ), procalcitonin (PCT) and high-sensitivity C-reactive protein (hs-CRP); SOFA, Sequential Organ Failure Assessment score; APECHE Ⅱ, Acute Physiology and Chronic Health Evaluation; AKI, Acute Kidney Injury.

For peer review only

知情同意书▪知情告知页

尊敬的先生/女士：

您好！您（或您的家属）当前所患的是□脓毒症□脓毒性休克。我们将邀请您（或您的家属）参加一项已上市药品治疗此疾病的临床研究：硫辛酸注射液治疗脓毒症及脓毒性休克成人患者的有效性及安全性的前瞻性、多中心、单盲、随机、安慰剂对照临床研究。该研究由茂名市人民医院、广东省人民医院发起。为了保证您的权益，本研究的研究方案和知情同意书均已获得伦理委员会的批准（编号：________________）。在同意参加这项研究之前，阅读并理解这份知情同意书对您来说十分重要。这份文件向您阐述了本研究的研究目的、步骤、可能给您带来的受益和风险等注意事项。请您仔细阅读，如有任何疑问请向负责该项研究的研究者提出。您参加本项研究是自愿的，并且可以在任何时间阶段退出研究。如果您决定参加这项研究，您将获得一份由双方签名的知情同意书副本。

一、研究背景与目的

脓毒症是机体对感染的反应失调而导致危及生命的器官功能障碍，脓毒性休克是指脓毒症合并出现严重的循环障碍和细胞代谢紊乱，是感染、烧/创伤、休克等急危重患者的严重并发症。脓毒症与脓毒性休克病死率高，大幅增加患者及家属经济负担。随着我国危重病监护救治技术的进步，脓毒症患者病死率虽然已显著下降，但仍高达 20~30%。针对脓毒症的病因治疗临床尚无特效药物。

研究表明，硫辛酸及其代谢产物是体内天然的生物抗氧化剂，鉴于氧化应激介导的细胞损伤是脓毒症重要病理生理学基础，通过捕捉体内自由基，从而抑制体内过度的炎症反应，改善脓毒症及脓毒性休克的治疗是可行的。我们拟在确诊脓毒症或脓毒性休克患者中应用硫辛酸治疗，探讨硫辛酸降低脓毒症及脓毒性休克患者病死率的有效性和安全性，以期降低患者病死率、提高救治成功率、缩短住院天数并改善改善预后。

二、研究内容和过程

本项研究采用前瞻性、多中心、单盲、随机、安慰剂对照临床研究设计，如果您符合本研究的入组标准，您将在脓毒症/脓毒性休克常规治疗（依据《拯救脓毒症和脓毒性休克运动指南》）或在常规治疗的基础上应用硫辛酸治疗，您分配在两种方案（硫辛酸组和安慰剂组）中接受治疗的概率是相同的。

本研究将记录您的个人情况和与疾病相关的临床资料：包括病史；常规医学检查（如血尿常规、肝肾功能、凝血功能、生化检查、血气分析、炎性因子和炎症标记物、影像学检查、

For peer review only - http://bmjopen.bmj.com/site/about/guidelines.xhtml

医学评分等）；为了客观评价病情变化，还将详细询问并记录您住院期间每天的病情变化，出院后随访至您入组后第 28 天。

以上治疗措施和医学检查项目，均为脓毒症和脓毒性休克诊断和治疗所必须的常规项目。本研究未设计超出常规诊断和治疗所必须的特殊检查和治疗项目，未增加患者的额外医疗费用。

三、参与本研究可能的受益

您和社会将可能从本研究中受益。此种受益包括您的病情有可能获得改善，减少器官损害的程度，硫辛酸免费用药，以及本研究可能帮助患有相似病情的其他患者。

无论您是否参加本研究，治疗与相关医学检查都会按本研究的常规方案进行。因此，参加本研究不会额外增加您的医疗负担；并且对您的病情观察和治疗将会更加全面、有利。

四、参与本研究可能的风险与不适

静脉滴注硫辛酸一般可能出现的不良反应有：例如头胀、呼吸困难、抽搐、复视、紫癜、出血倾向、过敏等。反应大多较轻且发生率较低，停药后可自行消失。

五、研究的保密性

如果您决定参加本项研究，您参加研究及在研究中的个人资料均属保密。您的血/尿等标本将以研究编号数字而非您的姓名加以标识。可以识别您身份的信息将不会透露给研究小组以外的成员，除非获得您的许可。所有的研究成员都被要求对您的身份保密。您的档案将保存在有锁的档案柜中，仅供研究人员查阅。为确保研究按照规定进行，必要时，政府管理部门或伦理委员会的成员按规定可以在研究单位查阅您的个人资料。这项研究结果发表时，将不会披露您个人的任何资料。

六、您的权利与义务

参加本研究期间需要您配合研究方案要求，接受研究医生对疾病预后的随访。

您可以选择不参加本项研究，或者在任何时候通知研究者要求退出研究，您做出的决定绝不影响您和医务人员的关系，您的任何医疗待遇、权益和医学关注不会因此受到影响，不会受到任何不公平的对待和惩罚。如果您决定退出研究，请您务必与您的研究医生联络，如果您在研究期间终止治疗和检查，本着对您的健康负责的目的，研究医生可能会询问一些与您健康相关的问题，还有可能要求您进行一些检查。

如果您需要其它治疗，或者没有遵循研究计划，或者您的主管研究医生认为您继续参与本研究不符合您的最大利益，研究医生可以让您退出研究；如果您在使用研究药物后出现不

For peer review only - http://bmjopen.bmj.com/site/about/guidelines.xhtml

适，或者安全风险，研究医生可能会在未征得您同意的情况下让您退出研究，研究医生将会与您讨论您退出研究后的相关事宜。

您可随时了解与本研究有关的信息资料和研究进展，如果您有与本研究有关的问题，或您在研究过程中发生了任何不适与损伤，或有关于本项研究参加者权益方面的问题您可以跟研究者联系。如果您对您作为研究参与者的权利有任何问题，您可以联系伦理委员会人员，电话：15915280880 。

致谢

医学科学的发展和进步离不开临床研究，您的参与将为医学科学进步做出贡献，对该疾病诊疗的研究与探索做出贡献。作为此项研究的研究者和申办者，我们将时刻铭记您的贡献，并对您表示最诚挚的感谢。

For peer review only

For peer review only - http://bmjopen.bmj.com/site/about/guidelines.xhtml

知情同意书▪同意签字页

在签署这份知情同意书前，我已经阅读上述信息，并且理解该项目的目的以及参加该项目可能带来的潜在利益和风险。我确认已经经过充分的考虑，也有机会对研究程序和研究药物提出疑问，并且所有的疑问均已得到令我满意的解答。

我同意研究医生收集和处理我的信息，包括与我健康有关的信息。我同意我的信息（个人信息除外）由研究单位处理。如果我决定退出本研究，我同意在此之前收集的信息仍可以被继续处理。

我有权在任何时候得到咨询服务，并有权决定在任何时候退出本研究计划而不会受到任何不利影响，不会因此丧失任何合法权利。我自愿签署这份知情同意书，并自愿参加此研究项目，会与研究者全面合作。我已得到此份文件的副本。

受试者

姓名(正楷) ____________________ 签名:__________________________

联系电话: _____________________ 日期: _____年____月____日

法定代理人/ 法定监护人

姓名(正楷) _________________ 签名:___________ 与受试者关系: _____________

联系电话: __________________ 日期: _____年____月____日

公正的见证人声明

我确认，同意书中的信息被进行了准确的解释，已经被患者和/或患者的法定代理人理解，同意意见是由患者和/或患者的法定代理人自愿提供的。

姓名(正楷) ____________________ 签名:__________________________

联系电话: _____________________ 日期: _____年____月____日

注：如果患者、法定监护人或法定代理人不能阅读及签署同意书（例如重度视力受损、诵读困难或不识字），必须有至少 1 名公正的见证人。公正的见证人在讨论知情同意书的整个过程中都必须在场。

我已准确地将知情同意书内容告知受试者并对受试者的提问进行了解答，受试者自愿参加本项临床研究。

研究者签名：_____________ 联系电话：________________

日期：____年____月____日

For peer review only

Reporting checklist for protocol of a clinical trial.

Based on the SPIRIT guidelines.

Instructions to authors

Complete this checklist by entering the page numbers from your manuscript where readers will find each of the items listed below.

Your article may not currently address all the items on the checklist. Please modify your text to include the missing information. If you are certain that an item does not apply, please write "n/a" and provide a short explanation.

Upload your completed checklist as an extra file when you submit to a journal.

In your methods section, say that you used the SPIRIT reporting guidelines, and cite them as:

Chan A-W, Tetzlaff JM, Gøtzsche PC, Altman DG, Mann H, Berlin J, Dickersin K, Hróbjartsson A, Schulz KF, Parulekar WR, Krleža-Jerić K, Laupacis A, Moher D. SPIRIT 2013 Explanation and Elaboration: Guidance for protocols of clinical trials. BMJ. 2013;346:e7586

		Reporting Item	Page Number
**Administrative information**			
Title	#1	Descriptive title identifying the study design, population, interventions, and, if applicable, trial acronym	1
Trial registration	#2a	Trial identifier and registry name. If not yet registered, name of intended registry	3
Trial registration: data set	#2b	All items from the World Health Organization Trial Registration Data Set	7
Protocol version	#3	Date and version identifier	n/a, no major amendments made
Funding	#4	Sources and types of financial, material, and other support	20

For peer review only

Roles and responsibilities: contributorship	#5a	Names, affiliations, and roles of protocol contributors	19
Roles and responsibilities: sponsor contact information	#5b	Name and contact information for the trial sponsor	n/a, it is a investigator initiated trial
Roles and responsibilities: sponsor and funder	#5c	Role of study sponsor and funders, if any, in study design; collection, management, analysis, and interpretation of data; writing of the report; and the decision to submit the report for publication, including whether they will have ultimate authority over any of these activities	20
Roles and responsibilities: committees	#5d	Composition, roles, and responsibilities of the coordinating centre, steering committee, endpoint adjudication committee, data management team, and other individuals or groups overseeing the trial, if applicable (see Item 21a for data monitoring committee)	13
**Introduction**			
Background and rationale	#6a	Description of research question and justification for undertaking the trial, including summary of relevant studies (published and unpublished) examining benefits and harms for each intervention	5-6
Background and rationale: choice of comparators	#6b	Explanation for choice of comparators	5-6
Objectives	#7	Specific objectives or hypotheses	9-11
Trial design	#8	Description of trial design including type of trial (eg, parallel group, crossover, factorial, single group), allocation ratio, and framework (eg, superiority, equivalence, non-inferiority, exploratory)	7, 12-13
**Methods: Participants,**			

For peer review only

**interventions, and outcomes**			
Study setting	#9	Description of study settings (eg, community clinic, academic hospital) and list of countries where data will be collected. Reference to where list of study sites can be obtained	7-8
Eligibility criteria	#10	Inclusion and exclusion criteria for participants. If applicable, eligibility criteria for study centres and individuals who will perform the interventions (eg, surgeons, psychotherapists)	9
Interventions: description	#11a	Interventions for each group with sufficient detail to allow replication, including how and when they will be administered	10
Interventions: modifications	#11b	Criteria for discontinuing or modifying allocated interventions for a given trial participant (eg, drug dose change in response to harms, participant request, or improving / worsening disease)	11
Interventions: adherance	#11c	Strategies to improve adherence to intervention protocols, and any procedures for monitoring adherence (eg, drug tablet return; laboratory tests)	n/a, adherence is not the issue in the settings of intensive care unit
Interventions: concomitant care	#11d	Relevant concomitant care and interventions that are permitted or prohibited during the trial	10
Outcomes	#12	Primary, secondary, and other outcomes, including the specific measurement variable (eg, systolic blood pressure), analysis metric (eg, change from baseline, final value, time to event), method of aggregation (eg, median, proportion), and time point for each outcome. Explanation of the clinical relevance of chosen efficacy and harm outcomes is strongly recommended	10-11
Participant timeline	#13	Time schedule of enrolment, interventions (including any run-ins and washouts), assessments, and visits for participants. A schematic diagram is highly	27-29

For peer review only

		recommended (see Figure)	
Sample size	#14	Estimated number of participants needed to achieve study objectives and how it was determined, including clinical and statistical assumptions supporting any sample size calculations	11-12
Recruitment	#15	Strategies for achieving adequate participant enrolment to reach target sample size	12
**Methods: Assignment of interventions (for controlled trials)**			
Allocation: sequence generation	#16a	Method of generating the allocation sequence (eg, computer-generated random numbers), and list of any factors for stratification. To reduce predictability of a random sequence, details of any planned restriction (eg, blocking) should be provided in a separate document that is unavailable to those who enrol participants or assign interventions	12-13
Allocation concealment mechanism	#16b	Mechanism of implementing the allocation sequence (eg, central telephone; sequentially numbered, opaque, sealed envelopes), describing any steps to conceal the sequence until interventions are assigned	12-13
Allocation: implementation	#16c	Who will generate the allocation sequence, who will enrol participants, and who will assign participants to interventions	12-13
Blinding (masking)	#17a	Who will be blinded after assignment to interventions (eg, trial participants, care providers, outcome assessors, data analysts), and how	13
Blinding (masking): emergency unblinding	#17b	If blinded, circumstances under which unblinding is permissible, and procedure for revealing a participant's allocated intervention during the trial	13
**Methods: Data collection, management, and analysis**			

For peer review only

Data collection plan	#18a	Plans for assessment and collection of outcome, baseline, and other trial data, including any related processes to promote data quality (eg, duplicate measurements, training of assessors) and a description of study instruments (eg, questionnaires, laboratory tests) along with their reliability and validity, if known. Reference to where data collection forms can be found, if not in the protocol	11
Data collection plan: retention	#18b	Plans to promote participant retention and complete follow-up, including list of any outcome data to be collected for participants who discontinue or deviate from intervention protocols	n/a, the follow-up window in our trial is a maximum of 28 days, which sees few lost to follow-up
Data management	#19	Plans for data entry, coding, security, and storage, including any related processes to promote data quality (eg, double data entry; range checks for data values). Reference to where details of data management procedures can be found, if not in the protocol	13
Statistics: outcomes	#20a	Statistical methods for analysing primary and secondary outcomes. Reference to where other details of the statistical analysis plan can be found, if not in the protocol	14-15
Statistics: additional analyses	#20b	Methods for any additional analyses (eg, subgroup and adjusted analyses)	n/a, subgroup and adjusted analyses is not planned
Statistics: analysis population and missing data	#20c	Definition of analysis population relating to protocol non-adherence (eg, as randomised analysis), and any statistical methods to handle missing data (eg, multiple imputation)	14

Methods: Monitoring

For peer review only

Data monitoring: formal committee	#21a	Composition of data monitoring committee (DMC); summary of its role and reporting structure; statement of whether it is independent from the sponsor and competing interests; and reference to where further details about its charter can be found, if not in the protocol. Alternatively, an explanation of why a DMC is not needed	13
Data monitoring: interim analysis	#21b	Description of any interim analyses and stopping guidelines, including who will have access to these interim results and make the final decision to terminate the trial	15
Harms	#22	Plans for collecting, assessing, reporting, and managing solicited and spontaneously reported adverse events and other unintended effects of trial interventions or trial conduct	10-11
Auditing	#23	Frequency and procedures for auditing trial conduct, if any, and whether the process will be independent from investigators and the sponsor	13
**Ethics and dissemination**			
Research ethics approval	#24	Plans for seeking research ethics committee / institutional review board (REC / IRB) approval	7
Protocol amendments	#25	Plans for communicating important protocol modifications (eg, changes to eligibility criteria, outcomes, analyses) to relevant parties (eg, investigators, REC / IRBs, trial participants, trial registries, journals, regulators)	7
Consent or assent	#26a	Who will obtain informed consent or assent from potential trial participants or authorised surrogates, and how (see Item 32)	12
Consent or assent: ancillary studies	#26b	Additional consent provisions for collection and use of participant data and biological specimens in ancillary studies, if applicable	12
Confidentiality	#27	How personal information about potential and enrolled	13

For peer review only

		participants will be collected, shared, and maintained in order to protect confidentiality before, during, and after the trial	
Declaration of interests	#28	Financial and other competing interests for principal investigators for the overall trial and each study site	20
Data access	#29	Statement of who will have access to the final trial dataset, and disclosure of contractual agreements that limit such access for investigators	13
Ancillary and post trial care	#30	Provisions, if any, for ancillary and post-trial care, and for compensation to those who suffer harm from trial participation	15
Dissemination policy: trial results	#31a	Plans for investigators and sponsor to communicate trial results to participants, healthcare professionals, the public, and other relevant groups (eg, via publication, reporting in results databases, or other data sharing arrangements), including any publication restrictions	15
Dissemination policy: authorship	#31b	Authorship eligibility guidelines and any intended use of professional writers	n/a, all authorship will be determined by the principal investigator according actual contributions
Dissemination policy: reproducible research	#31c	Plans, if any, for granting public access to the full protocol, participant-level dataset, and statistical code	15
**Appendices**			
Informed consent materials	#32	Model consent form and other related documentation given to participants and authorised surrogates	12
Biological specimens	#33	Plans for collection, laboratory evaluation, and storage of biological specimens for genetic or molecular analysis in the current trial and for future use in	n/a, blood specimens will be

For peer review only

	ancillary studies, if applicable	simultaneously collected and tested in hospital clinical laboratory as routine

None The SPIRIT Explanation and Elaboration paper is distributed under the terms of the Creative Commons Attribution License CC-BY-NC. This checklist can be completed online using https://www.goodreports.org/, a tool made by the EQUATOR Network in collaboration with Penelope.ai

For peer review only

Adjuvant Lipoic acid Injection in Sepsis treatment in China (ALIS study): protocol for a randomized, single-blind, placebo-controlled trial

Journal:	*BMJ Open*
Manuscript ID	bmjopen-2023-072897.R1
Article Type:	Protocol
Date Submitted by the Author:	10-Jul-2023
Complete List of Authors:	Hu, Linhui; Maoming People's Hospital, Department of Critical Care Medicine; Maoming People's Hospital, Clinical Research Center, Center of Scientific Research,<br>Zhou, Xinjuan; Maoming People's Hospital, Clinical Research Center, Center of Scientific Research; Maoming People's Hospital, Department of Cardiology<br>Huang, Jinbo; Maoming People's Hospital, Clinical Research Center, Center of Scientific Research<br>He, Yuemei; Maoming People's Hospital, Clinical Research Center, Center of Scientific Research; Maoming People's Hospital, Department of Cardiology<br>Wu, Quanzhong; Maoming People's Hospital, Department of Surgery Intensive Care Medicine<br>Huang, Xiangwei; Maoming People's Hospital, Department of Critical Care Medicine<br>Wu, Kunyong; Maoming People's Hospital, Center of Scientific Research<br>Wang, Guangwen; Maoming People's Hospital, Center of Scientific Research<br>Li, Sinian; Maoming People's Hospital, Department of Neurocritical Care Unit<br>Chen, Xiangyin; Maoming People's Hospital, Department of Surgery Intensive Care Medicine<br>Chen, Chunbo; Shenzhen People's Hospital, Department of Critical Care Medicine
**Primary Subject Heading**:	Intensive care
Secondary Subject Heading:	Intensive care
Keywords:	Clinical trials < THERAPEUTICS, Adult intensive & critical care < INTENSIVE & CRITICAL CARE, Randomized Controlled Trial, Protocols & guidelines < HEALTH SERVICES ADMINISTRATION & MANAGEMENT

Adjuvant Lipoic acid Injection in Sepsis treatment in China (ALIS study): protocol for a randomized, single-blind, placebo-controlled trial

Linhui Hu[1,2, †], Xinjuan Zhou[2,3 †], Jinbo Huang[2, †], Yuemei He[2,3 †], Quanzhong Wu[4, †], Xiangwei Huang[1], Kunyong Wu[5], Guangwen Wang[5], Sinian Li[6], Xiangyin Chen[4], Chunbo Chen[7*]

[1]Department of Critical Care Medicine, Maoming People's Hospital, 101 Weimin Road, Maoming, 525000, Guangdong, China.

[2]Clinical Research Center, Center of Scientific Research, Maoming People's Hospital, 101 Weimin Road, Maoming, 525000, Guangdong, China.

[3]Department of Cardiology, Maoming People's Hospital, Maoming, China.

[4]Department of Surgery Intensive Care Medicine, Maoming People's Hospital, 101 Weimin Road, Maoming, 525000, Guangdong, China.

[5]Center of Scientific Research, Maoming People's Hospital, Maoming, 101 Weimin Road, Maoming, 525000, Guangdong, China.

[6]Department of Neurocritical Care Unit, Maoming People's Hospital, 101 Weimin Road, Maoming, 525000, Guangdong, China.

[7]Department of Critical Care Medicine, Shenzhen People's Hospital, The Second Clinical Medical College of Jinan University, The First Affiliated Hospital of Southern University of Science and Technology, No.1017 Dongmen North Road, Shenzhen 518000, Guangdong, China.

†These five authors contributed equally to this study as co-first authors.

*Corresponding author:

Chunbo Chen, gghccm@163.com

Department of Critical Care Medicine, Shenzhen People's Hospital, The Second Clinical Medical College of Jinan University, The First Affiliated Hospital of

For peer review only

Southern University of Science and Technology, No.1017 Dongmen North Road, Shenzhen 518000, Guangdong, China

Email addresses for all authors:

L.H.: hulinhui@live.cn

X.Z.: z1328314459@dingtalk.com

J.H.: 2589618856@qq.com

Y.H.: hym202008@163.com

Q.W.: wqz196@163.com

X.H.: 13828681930@163.com

K.W.: 237147873@qq.com

G.W.: 929233133@qq.com

S.L.: lisinian135455@163.com

X.C.: xychen569@163.com

C.C.: gghccm@163.com

For peer review only

ABSTRACT

Introduction Sepsis is a life-threatening immune disorder resulting from an dysregulated host response to infection. Adjuvant therapy is a valuable complement to sepsis treatment. Lipoic acid has shown potential in attenuating sepsis-induced immune dysfunction and organ injury in vivo and in vitro studies. However, clinical evidence of lipoic acid injection in sepsis treatment is lacking. Hence, we devised a randomized controlled trial to evaluate the efficacy and safety of lipoic acid injection in improving the prognosis of sepsis or septic shock patients.

Methods and analysis A total of 352 sepsis patients are planned to be recruited from intensive care units (ICUs) at eight tertiary hospitals in China for this trial. Eligible participants will undergo randomization in a 1:1 ratio, allocating them to either the control group or the experimental group. Both groups received routine care, with the experimental group also receiving lipoic acid injection and the control group receiving placebo. The primary efficacy endpoint is 28-day all-cause mortality. The secondary efficacy endpoints are: ICU and hospital mortality, ICU and hospital stay, new acute kidney injury in ICU, demand and duration of life support, Sequential Organ Failure Assessment (SOFA)/Acute Physiology and Chronic Health Evaluation II (APACHE II) and changes from baseline (ΔSOFA/ΔApache II), arterial blood lactate (LAC) and changes from baseline (ΔLAC), blood procalcitonin (PCT), high-sensitivity C-reactive Protein (hs-CRP), interleukin-2 (IL-2), IL-4, IL-6, IL-10, tumor necrosis factor-α (TNF-α), and interferon-γ (IFN-γ) and changes from baseline on Day 1 (D1), D3, D5, and D7. Clinical safety will be assessed through analysis of adverse events.

Ethics and dissemination The study was approved by the Ethics Committee of Maoming People's Hospital (approval no. PJ2020MI-019-01). Informed consent will be obtained from the participants or representatives. The findings will be disseminated through academic conferences or journal publications.

Trial registration ChiCTR2000039023.

Strengths and limitations of this study

- The trial utilizes a randomized controlled design with a substantial sample size.
- The trial design mitigates the influence of confounding factors, such as terminal diseases.
- Adequate evidence from animal and human research supports the hypothesis that lipoic acid could protect organs against sepsis injury.
- This trial follows a single-blind study design rather than a double-blind design.

For peer review only

INTRODUCTION

Sepsis, characterized by acute organ dysfunction resulting from a dysregulated host response to infection, is a multifaceted disorder that poses a significant mortality risk among patients in non-coronary intensive care units (ICUs) [1-3]. Despite the advancements in sepsis management over the past decade, the mortality rate still exceeds 30%, reaching up to 60% in cases of septic shock [4]. In high-income countries, the population incidence rate of sepsis was 437 cases per 100,000 person-years, resulting in approximately 2.8 million deaths attributed to sepsis annually [5]. Du et al. [6] reported a standardized sepsis incidence and mortality rate of 461 and 79 cases per 100,000 person-years respectively in China. The incidence is expected to rise further due to the aging Chinese population, leading to an increased financial burden associated with treatment. Consequently, prioritizing the management of sepsis and septic shock becomes crucial for ensuring patient safety.

The primary treatment approach for sepsis emphasizes timely intervention, which includes removing the source of infection, administering appropriate antibiotics, and restoring tissue perfusion through fluid resuscitation and vasoactive drugs [7-9]. Indeed, sepsis is a syndrome rather than a specific disease, which highlights the importance of implementing adjuvant therapies that target its underlying pathophysiological mechanisms beyond primary treatment. The 2021 guidelines only provide weak recommendations for adjuvant therapies in sepsis management [10], such as the use of intravenous corticosteroids. Consequently, there is a pressing need for safe, effective, and affordable adjuvant treatments with minimal adverse effects.

The exact mechanisms of sepsis-induced cell injury and organ dysfunction are not fully understood. However, it is widely recognized that simultaneous imbalanced activation of pro-inflammatory response (leading to cellular and tissue damage) and anti-inflammatory response (leading to immune system impairment) are associated with sepsis [11-13]. Moreover, sepsis progression has been linked to the imbalance between reactive oxygen species (ROS) and antioxidant enzymes. One compound that has shown unique antioxidant properties is lipoic acid (LA), also known as 1,2-dithiolane-3-pentanoic acid. LA is a naturally occurring dithiol compound that is

For peer review only

enzymatically synthesized in the mitochondria from octanoic acid. LA chelates several metal ions [14] and scavenges ROS and reactive nitrogen species (RNS) both in vitro and in vivo [15]. These actions enable LA to regenerate antioxidant molecules like glutathione, vitamin C, and vitamin E [16]. Remarkably, due to its small chemical structure, lipoic acid (LA) exhibits solubility in both water and lipids. This unique characteristic enables LA to efficiently access and permeate throughout the entire body, facilitating its favorable effects.

In a study conducted by Zhang et al. [17], it was demonstrated that LA effectively attenuates lipopolysaccharide (LPS)-induced acute inflammatory responses both in vitro and in vivo. This attenuation is achieved through the activation of the phosphoinositide 3-kinase (PI3K)/protein kinase B (Akt) signaling pathway. Multiple studies [18-21] have demonstrated that LA possesses anti-inflammatory properties and can effectively reduce LPS-induced inflammation. LA achieves this by inhibiting the activation of the nuclear factor κB (NF-κB) signaling pathway and suppressing the excessive production of pro-inflammatory factors. These actions contribute to the attenuation of sepsis-induced inflammation and oxidative damage in organs, highlighting the potential of LA as a therapeutic intervention for sepsis. Furthermore, LA has shown significant benefits in improving diabetic neurovascular and metabolic abnormalities [22]. Additionally, it has been implicated in cardiovascular protection and exerting anti-inflammatory effects [23-26]. This suggests that LA may have broad therapeutic applications beyond sepsis treatment. Considering its anti-oxidative properties, LA holds promise as a potential adjuvant for sepsis treatment. Its ability to protect tissues from oxidative stress and prevent sepsis-induced organ dysfunction makes it a candidate worth exploring further.

Indeed, LA has been investigated in various therapeutic contexts, including weight management[27, 28], multiple sclerosis [29], male fertility [30], and type 2 diabetes [31, 32], showing promising results. However, when it comes to sepsis-induced organ dysfunction, most studies have primarily focused on animal models, particularly rats [17, 33, 34]. As of today, there is a lack of human studies exploring the therapeutic effects of LA in sepsis treatment.

For peer review only

To address this gap in knowledge, we have designed a multicenter, single-blinded, randomized controlled trial (RCT) in China. The objective of this trial is to assess the efficacy and safety of LA injection compared to a placebo in patients diagnosed with sepsis or septic shock in intensive care units (ICUs). We aim to investigate the preventive and therapeutic effects of LA on suppressing inflammation, attenuating organ dysfunction, shortening ICU or hospital stay, and improving patient outcomes, including mortality. By conducting this RCT, we hope to provide valuable insights into the potential benefits of LA in sepsis treatment.

METHODS AND ANALYSIS

Study design and setting

The ALIS ("Adjuvant Lipoic Acid Injection in Sepsis Treatment in China") study is a multicenter, single-blind, randomized, placebo-controlled clinical trial conducted in tertiary hospitals in China. The protocol and informed consent forms are required undergo thorough review and approval by the applicable ethical committees (ECs) to ensure compliance with scientific standards and human subject protection regulations at each participating center. The study involves various departments, including general ICU, neurological ICU, surgical ICU, and emergency ICU. Detailed information about the intended participating centers can be found in Table 1. The enrollment period for this study is estimated to be two years, and the trial will conclude with the last follow-up. The study has been registered on chictr.org.cn (ChiCTR2000039023) [35], ensuring transparency and accessibility of the study information. The study design and protocol adhere to the Standard Protocol Items: Recommendations for Interventional Trials (SPIRIT) reporting guidelines [36].

Protocol amendments

Protocol amendments will be recorded, including a concise description of the modification and a reference (date and number), in cases where changes to the existing protocol have a significant impact on subject safety, study scope, or scientific

For peer review only

quality. The coordinating investigator has a responsibility to inform all investigators, the reviewing ethical committee, and other relevant parties about these protocol amendments as required.

Table 1. Research settings and names of each ethics committee

Research setting	Ethics committee name	Hospital rank and location
Maoming People's Hospital	Medical Ethics Committee of Maoming People's Hospital	Tertiary, Maoming, Guangdong, China
Guangdong Provincial People's Hospital	Medical Ethics Committee of Guangdong Provincial People's Hospital	Tertiary, Guangzhou, Guangdong, China
Affiliated Hospital of Guangdong Medical university	Medical Ethics Committee of Affiliated Hospital of Guangdong medical university	Tertiary, Zhanjiang, Guangdong, China
Central People's Hospital of Zhanjiang	Medical Ethics Committee of Central People's Hospital of Zhanjiang	Tertiary, Zhanjiang, Guangdong, China
Huizhou Central Hospital	Medical Ethics Committee of Huizhou Central Hospital	Tertiary, Huizhou, Guangdong, China
Xiaolan People's Hospital of Zhongshan City	Medical Ethics Committee of Xiaolan People's Hospital of Zhongshan	Tertiary, Zhongshan, Guangdong, China
Third People's Hospital of Huizhou	Medical Ethics Committee of the Third People's Hospital of Huizhou	Tertiary, Huizhou, Guangdong, China
Yunfu People's Hospital	Medical Ethics Committee of Yunfu People's Hospital	Tertiary, Yunfu, Guangdong, China

For peer review only

Eligibility criteria

All trial centers will adhere to the same participant inclusion criteria. The inclusion criteria are as follow:

1. Meeting the diagnostic criteria for sepsis and septic shock, as established by the American Critical Care Medical Association/European Critical Care Medical Association (Sepsis-3 criteria) [37].
2. Age between 18 and 80 years.
3. Within 48 hours of receiving a diagnosis of sepsis or septic shock.
4. Having provided signed informed consent.

The exclusion criteria for participant selection are as follows:

1. Female patients who are pregnant or lactating.
2. Presence of persistent infection sources that cannot be effectively eliminated through procedures such as puncture, drainage, debridement, or other surgical operations.
3. Participation in another clinical trial.
4. Current treatment with other medications that have antioxidant effects, such as vitamin C and vitamin E.
5. Accompanied by non-infectious but life-threatening conditions, such as advanced tumor, severe craniocerebral injury, uncontrollable massive bleeding, cardiogenic shock, etc.
6. Lack of approval for comprehensive and active life support treatment.
7. Allergy to LA or similar active agents (e.g., Vitamin B), or previous intolerance to the recommended dose of LA.

Interventions

The participants will be randomly assigned to either the placebo group or the LA group and treated according to their assigned treatment plan. In the placebo group, patients will receive a placebo consisting of 250 ml of normal saline. In the treatment group, patients will receive a solution containing an LA injection of 600mg (provided by Yabao Pharmaceutical Group Co., Ltd.) dissolved in 250 ml of normal saline. To

For peer review only

prevent exposure to light, both the LA and the placebo solutions will be wrapped in aluminum platinum paper. It is important that the intravenous drip time does not fall short of 30 minutes. Concomitant care include standard sepsis bundle treatment and other routine care. The critical care physicians are encouraged to adhere to the updated Surviving Sepsis Campaign guidelines [7], although the final decision is at the discretion of the physicians as per institutional guidelines. The number and percentage of patients in both the treatment and placebo groups who deviated from the sepsis bundle treatment will be documented.

Evaluation and outcomes

Participants in the study will undergo clinical evaluation and laboratory testing, as outlined in Supplementary Table 1. The collected data will include demographic information such as age and gender, disease diagnosis and progression, interventions and prognosis, as well as adverse events (AEs). The disease diagnosis data will encompass primary and concomitant diseases, blood culture results, white blood cell count, and liver and kidney biochemistry. Disease progression data will involve baseline severity scores and serum biomarkers, including Sequential Organ Failure Assessment (SOFA) score, Acute Physiology and Chronic Health Evaluation II (APACHE II) score, Procalcitonin (PCT), high-sensitivity C-reactive protein (hs-CRP), Interleukin 2 (IL-2), IL-4, IL-6, IL-10, Tumor Necrosis Factor-α (TNF-α), and Interferon-γ (IFN-γ). Follow-up values on specific days (Day 1 [D1], D3, D5, D7) after randomization will also be recorded, along with the changes from baseline.

Data related to disease intervention will include the requirement and duration of life support measures like mechanical ventilation, vasoactive drugs, and continuous renal replacement therapy. Disease prognosis data will encompass 28-day all-cause mortality, ICU mortality, in-hospital mortality, incidence of new-onset acute kidney injury (AKI) after ICU admission, ICU stay, and length of hospital stay. Throughout the study, data on organ dysfunction, occurrence of AKI, and safety events will be continuously monitored.

For peer review only

The primary efficacy endpoint of the study is 28-day all-cause mortality. The secondary efficacy endpoints are:

1. ICU mortality
2. Hospital mortality
3. Duration of ICU and hospital stay
4. Incidence of new acute kidney injury in ICU
5. SOFA/APACHE II scores on specific days (D1, D3, D5, and D7) and their changes from baseline (ΔSOFA/ΔApache II)
6. Demand and duration of life support measures (vasopressors, invasive mechanical ventilation, and continuous renal replacement therapy)
7. Lactate concentration of arterial blood on specific days (D1, D3, D5, and D7) and their changes from baseline (ΔLAC)
8. Levels of PCT, hs-CRP, IL-2, IL-4, IL-6, IL-10, TNF-α, and IFN-γ on specific days (D1, D3, D5, and D7) and their changes from baseline.

The detection of levels of IL-2, IL-4, IL-6, IL-10, TNF-α, and IFN-γ is not mandatory if unavailable.

The safety endpoint of the study includes the assessment of any adverse events (AEs), including serious adverse events (SAEs) and adverse drug reactions (ADRs). These ADRs may manifest as various symptoms, such as head swelling, dyspnea, convulsion, diplopia, purpura, bleeding tendency, allergies, and others.

During clinical research, it is essential for researchers to closely observe any AEs experienced by subjects. When AEs are identified, researchers should promptly take appropriate measures based on the subjects' conditions. Regardless of whether the AEs are associated with LA injection or not, it is necessary for researchers to diligently record them in detail within the case report form. The recorded information regarding AEs should encompass a description of the events, their occurrence time, severity, duration, measures taken, final results, and outcomes. These records serve the purpose of analyzing the potential correlation between AEs and LA injection, and they need to be signed and dated. In the event of encountering any SAEs, it is mandatory to report them to the principal investigator of the trial, the ethics

For peer review only

committee, and the state supervision agency within 24 hours of their occurrence. When an AE is identified, the researcher can administer necessary treatment measures considering the subject's condition, such as dose adjustment or temporary suspension of research drug usage. The decision regarding whether to terminate the study will depend on the circumstances. In case of an SAE occurrence, immediate action must be taken by the researcher to safeguard the safety of the subjects.

Participant timeline

The follow-up period for this trial is 28 days. At the final visit of the last patient, data regarding the clinical status of all participants who have either completed the trial or withdrawn will be collected.

Sample size

In this study, the primary efficacy endpoint was the 28-day all-cause mortality rate. A large-scale epidemiological survey conducted in the ICU revealed that the control group had a 38.2% 28-day all-cause mortality rate. In an RCT comparing the LA injection treatment group, the relative risk reduction was approximately 0.33 [38]. The anticipated 28-day all-cause mortality rate for the treatment group was expected to be 22.9% with a one-sided test, using $\alpha = 0.025$ and $\beta = 0.2$ (power = 80%). The threshold value, Δ, was set at 0.

Participants are randomized at a ratio of 1:1, resulting in a calculated sample size of 160 for each group using a superiority test (where a lower rate is considered superior). Taking into account the anticipated dropout rate of around 10%, the sample size was increased to 176 cases in each group.

Recruitment

To achieve adequate participant enrollment and reach the target sample size, the trial is planned to be conducted in multiple tertiary hospitals as outlined in Table 1. The enrollment period is estimated to span two years. The study successfully recruited its first participant on August 26, 2021. A well trained study coordinator has been

For peer review only

appointed at each participating center to meticulously screen all potentially eligible patients based on the predefined eligibility criteria. The anticipated timeline for completion of the study is set for September 1, 2024.

Consent and assent

The clinical investigator has the responsibility to obtain informed consent from every research subject prior to their participation in the study. Detailed information about the informed consent can be found in Appendix 1. In cases where a research subject is unable to provide consent due to a critical illness or disability, informed consent is obtained from authorized surrogates. However, it is important to note that obtaining prior approval from the Ethics Committee (EC) is necessary for this process. Our intention is solely to obtain consent for the use of data and samples related to the research question outlined in this protocol. We do not have any plans to utilize participant data or biological samples for ancillary studies.

Allocation

In this study, participants who have been diagnosed with sepsis based on the Sepsis-3 criteria are included in the screening process. Those who meet the eligibility criteria will be randomly assigned to either the treatment or control group using a 1:1 allocation. The randomization will be conducted using a computer-generated block randomization schedule with a block size of 8, stratified by site. Each block will consist of four patients receiving the LA injection and four patients receiving a placebo. The randomization method and block size will not be blinded until all data analysis is completed. The investigators involved in the study will not participate in data collection. The randomization center, through net verification, will assign the screened patients at each hospital in a random manner.

To ensure the overall quality and integrity of the clinical trial, code breaks should only occur in exceptional circumstances when knowledge of the actual treatment is absolutely necessary for further patient management. Investigators are encouraged to consult with medical advisors if they believe that unblinding is necessary. Any

For peer review only

instances of code breaks must be reported, along with the reason, on the corresponding case report form (CRF) page. It is important to note that unblinding should not automatically result in the discontinuation of the study drug.

Data collection and management

To ensure the competence in managing the project, data collectors, researchers, and coordinators involved in the study will receive thorough training in advance. The coordinators at each center will utilize an electronic data acquisition system for inputting the case report form (CRF) information. The specific system being used has obtained copyright for its computer software on April 6, 2022 (Software No. 9387742). After data entry, a designated individual will regularly perform accuracy checks to ensure the integrity of the entered data. If any data revisions are necessary, the modified data will undergo review by the data manager. Data access will be strictly restricted to authorized personnel only. The principal investigators at each center will have the ability to log into the system and view the data, but access to full information will only be granted with permission from the coordinating investigators of the research. It is emphasized that all research-related documents will be treated as highly confidential and stored securely at the study site.

Data monitoring

An independent Data and Safety Monitoring Board (DSMB) will assume responsibility for data monitoring and blinded analysis in this trial. DSMB committee members will regularly review the research data to ensure its integrity. Furthermore, the trial will undergo examination by the medical ethics committee of each participating center, which will assess its adherence to ethical principles and guidelines.

Analyses sets

The analysis of the trial will be conducted following the "intention to treat" principle. This means that all patients who have undergone randomization, regardless of

For peer review only

whether they completed the trial or received the designated treatment, will be included in the analysis according to their original assigned groups. The randomization information will be retained to the maximum extent possible, ensuring that the integrity of the randomization process is maintained during the analysis.

Intention-to-treat set (ITTS)

The principle of intention-to-treat (ITT) analysis is adopted to retain the randomized information effectively and attribute differences in trial results to treatment variations. This ensures that the assessment of treatment effects, specifically in the case of LA treatment, is conducted in the most accurate manner possible. The ITT set (ITTS) consists of all patients who were randomized, regardless of whether they received their designated treatment or completed the full trial. All patients in the ITTS remain in their original assigned groups for analysis. By adhering to the ITT principles, the analysis maintains the integrity of the randomization process and provides a comprehensive evaluation of the treatment's effectiveness, accounting for the real-world scenario.

Per protocol set (PPS)

The PPS includes all participants who meet the eligibility criteria and receive treatment as per their assigned randomization. These participants demonstrate good compliance with the trial protocol, including being prepared to receive treatment, undergoing measurements for primary efficacy endpoints, and ensuring that the necessary information is filled in the Case Report Form (CRF). The PPS is specifically utilized to analyze the primary efficacy endpoints of the study. By focusing on participants who adhere closely to the trial protocol, the PPS analysis provides insights into how the treatment performs under optimal conditions and can help evaluate the treatment's efficacy in a controlled setting.

For peer review only

Security set (SS)

The SS comprises all actual cases that have received at least one administration of the assigned fluid as per randomization. This set includes participants for whom safety endpoints are recorded, allowing for the assessment of adverse events. The incidence of adverse events is calculated based on the number of patients included in the safety set. By analyzing the safety data from this set, researchers can evaluate the occurrence and severity of adverse events related to the administered fluid, providing important insights into the safety profile of the treatment.

Statistical analysis

All analyses, including the primary analyses, will be conducted in accordance with the ITT principle. The data will be analyzed using R software v3.3.3 (R Foundation for Statistical Computing, Vienna, Austria) and RStudio v1.0.136 (RStudio Inc, Boston, MA, USA). Descriptive statistics will be used to describe the data's distribution, with continuous data presented as mean (standard deviation) or median and interquartile range, depending on the data characteristics. Categorical data will be reported as number and percentage. Group comparisons will be conducted using group t-tests or ANOVA for continuous data assuming normality, and non-parametric tests will be used if normality assumptions are not met. Categorical results will be compared using χ^2 test or Fisher's exact test. The statistical significance level for all tests will be set at a p-value of less than 0.05.

Interim analysis

No interim analysis is scheduled for this trial.

Ancillary and post-trial care

In each participating study center, all patients will receive treatment, monitoring, and routine assessments related to the recovery process in accordance with the Surviving Sepsis Campaign guidelines.

For peer review only

Patient and public involvement

None.

ETHICS AND DISSEMINATION

Ethics approval

This study has received approval from the Ethics Committee of Maoming People's Hospital, China. The approval (NO. PJ2020MI-019-01) was granted on September 25, 2020. Prior to enrollment in the study, all participants will be required to provide informed consent that has been approved by the ethics committee. Once enrolled, the study will be conducted in strict adherence to the clinical research plan and in accordance with the principles outlined in the Declaration of Helsinki.

Informed consent forms and privacy protection

In this study, individuals will not be eligible to participate in the study until they have obtained a signed and written informed consent form. Throughout the participants' involvement in the study, any updated versions of the informed consent forms and written information will be provided to them. The informed consent forms are considered important documents of the clinical research and will be retained for future reference. If any changes are made to the informed consent form during the study, the revised version can only be used after obtaining written approval from the ethics committee, and participants will be required to re-sign the revised version.

Participants in this project acknowledge and endorse the guidelines established to safeguard the privacy of subjects from any infringement. Throughout the entire duration of the study, the original data of the participants will only be linked to the clinical research database or files of the participating units using a unique identification number. Limited subject characteristics, such as gender, age, date of birth, and initials of the subject's name, may be used to verify the accuracy of the subject's information and their unique identification number, with the approval of

For peer review only

applicable laws and regulations. These measures ensure the confidentiality and protection of the participants' data.

Dissemination

The results of this study will be shared through presentations at relevant national and international conferences. Additionally, the findings will be submitted for publication in peer-reviewed journals. This dissemination approach ensures that the research outcomes reach a wide audience and undergo rigorous evaluation by experts in the field.

DISCUSSION

Sepsis is a severe medical condition characterized by dysfunction of organs caused by the body's uncontrolled response to an infection. It is defined by an acute increase in the Sequential Organ Failure Assessment (SOFA) score of 2 points or more [39]. Septic shock, on the other hand, is a subset of sepsis. It refers to a condition where there are profound circulatory, cellular, and metabolic abnormalities that result in hypotension, requiring the administration of vasopressor medications to maintain a mean arterial blood pressure above 65 mmHg. In septic shock, patients also exhibit elevated levels of serum lactate concentration, exceeding 2 mmol/L, despite adequate fluid resuscitation efforts.

In 2017, the World Health Assembly recognized sepsis and septic shock as global health priorities due to their high prevalence and alarming mortality rates [40]. Moreover, many sepsis survivors suffer from long-term complications, underscoring the urgent need to address this issue. While most data on sepsis come from high-income countries, where 2.8 million deaths are attributed to sepsis annually, a study conducted by Du et al. [6] reported standardized incidence and mortality rates of 461 and 79 cases per 100,000 person-years, respectively, in China. However, it is plausible that these numbers might be higher due to lower awareness of sepsis and limited access to intensive care and organ support. Despite being a global priority, the available research and evidence on sepsis disproportionately come from high-income

For peer review only

countries. Therefore, there is a critical need for more research, particularly in low-income and middle-income countries like China, which has a population exceeding 1.4 billion. This research should focus on identifying pharmacological interventions that can improve outcomes by targeting the underlying pathophysiological mechanisms of the disease.

Sepsis is characterized by a dysregulated systemic host response to infection. One well-established pathway of immune activation in response to infection involves the recognition of highly conserved microbial pathogen-associated molecular patterns by pattern-recognition receptors, including Toll-like receptors, present on innate immune system cells. This recognition leads to the activation of NF-κB and neutrophils, triggering the release of pro-inflammatory and anti-inflammatory mediators. Cytokines such as TNF-α, IL-1, IL-2, IL-6, IL-8, among others, promote neutrophil-endothelial cell adhesion and activate the complement and clotting cascades, contributing to what is commonly referred to as the "inflammatory storm".

The PI3K/Akt pathway is a well-conserved family of signal transduction enzymes that plays a crucial role in regulating cellular proliferation and survival. However, there are notable variations in the anti-inflammatory function of the PI3K/Akt pathway across different cell types. Depending on the specific stimulus and cell type involved, it can act as either a positive [41-43] or negative regulator [44-46] of NF-κB activation and cytokine production. Indeed, an increasing body of evidence indicates that the PI3K/Akt pathway serves as a critical negative feedback regulator of exaggerated innate immune and Toll-like receptor-mediated pro-inflammatory responses [44-51]. Multiple studies [44, 47-49, 52, 53] support the role of the PI3K/Akt pathway in limiting inflammation in endotoxemia and sepsis. Inhibition of PI3K/Akt signaling has been found to enhance LPS-induced activation of NF-κB and AP-1, along with increased gene expression of TNF-α and tissue factor in cultured cells [44]. NF-κB has been extensively documented to play a significant role in the transcriptional regulation of inflammatory genes induced by LPS, which contribute to the progression of septic shock, multiple organ failure, and mortality [54-57]. Clinically, increased NF-κB binding activity has been observed in patients with acute

For peer review only

inflammation and sepsis, demonstrating a correlation with clinical severity and mortality [58, 59]. As a result, LA could potentially suppress septic inflammation by modulating the PI3K/Akt pathway.

Oxidative stress, a significant promoter and mediator of the systemic inflammatory response, is considered crucial in the pathophysiology of sepsis [60]. The early phase of sepsis is characterized by an excessive formation of ROS and RNS, including superoxide and nitric oxide. Excessive production of these radical species during inflammation is associated with sepsis development, leading to marked oxidative stress, cellular injury, disruption of antioxidant systems, and mitochondrial dysfunction, ultimately resulting in organ damage [61, 62]. In normal conditions, a complex network of antioxidant defenses can counteract oxidative stress and protect mitochondria. However, severe oxidative stress occurs in sepsis, overwhelming the host's endogenous antioxidant defenses and disrupting cellular redox balance. It has been proposed that targeted antioxidant therapy aimed at mitochondria, which enhances endogenous antioxidant defenses, holds potential for protecting against radical-induced injury [63, 64]. Thus, LA may be beneficial in preventing organ injury by acting as a suppressor of ROS.

In summary, LA, as an agent that modulates inflammatory cascades through the aforementioned mechanisms, shows potential effectiveness in balancing the immune response and reducing organ impairment in septic patients. Therefore, we have developed a study protocol for an RCT to investigate the efficacy and safety of LA injection as a treatment for sepsis in China.

This study design possesses several strengths. Firstly, the utilization of a large sample size in this RCT can enhance our understanding of the efficacy of LA, a commonly used adjuvant drug in sepsis treatment lacking evidence-based support. Secondly, the exclusion of terminal disease as a confounding factor is addressed in this study. Sepsis can occur in patients already succumbing to other causes (e.g., terminal cancer), and inherent mortality associated with sepsis is a characteristic of the syndrome itself. Thirdly, the hypothesis that LA provides organ protection against sepsis injury is backed by substantial evidence from both animal and human research.

For peer review only

Nonetheless, there are certain limitations that should be acknowledged. Firstly, the relatively limited number of participating centers may introduce selection bias, potentially affecting the generalizability of the research findings to other contexts or settings. Secondly, this study design adopts a single-blind approach, which could introduce diagnostic bias by the investigators, potentially resulting in treatment imbalances between the control and treatment groups.

In conclusion, the ALIS study is a large-sample-sized, multicenter, single-blinded, randomized, parallel-group, placebo-controlled trial in China. It is designed based on robust evidence from basic and clinical studies. The anticipated outcomes of this trial have the potential to offer evidence-based recommendations for sepsis prevention in critically ill patients.

Contributors

CBC conceived of the study. LHH, XJZ, JBH, YMH, and QZW initiated the study design and XWH, KYW, GWW, SNL and XYC helped with implementation. CBC and LHH are grant holders. LHH, YMH and XJZ provided statistical expertise in clinical trial design. LHH and XJZ drafted the manuscript. CBC helped draft and revise the manuscript. All authors contributed to refinement of the study protocol and approved the final manuscript.

Funding

Author Chunbo Chen is currently receiving a grant (#MaoRenCaiBan [2020]24) from the Office of Talent Work Leading Team in Maoming, a grant (#201803010058) from the Guangzhou Science and Technology Program, a grant (#82172162) from the National Natural Science Foundation of China, and a grant (#DFJH2020028) under the Major Program of Summit Project, Guangdong Province High-level Hospital Construction Project of Guangdong Provincial People's Hospital, Guangdong Academy of Medical Sciences. Author Linhui Hu is currently receiving a grant (#2020YJ01) from the Emergent Science and Technology Project for Prevention and

For peer review only

Treatment of Novel Coronavirus Pneumonia, a grant (#zx2020017) from the High-level Hospital Construction Research Project of Maoming People's Hospital, and a grant (#SY2021005) from Excellent Young Talents Project of Maoming People's Hospital, a grant (#B2022246) from Medical Research Fund of Guangdong Province, and a grant (#2021KJZXZJYX003) from Special Science and Technology Fund of Maoming City. The study was supported by the Special Science and Technology Fund of Maoming City. The funders had no role in the design and conduct of the study; collection, management, analysis, and interpretation of the data; preparation, writing, review, or approval of the report; and decision to submit the report for publication.

Competing interests

The authors declare that they have no competing interests.

Patient consent for publication

Not applicable.

Provenance and peer review

Not commissioned; externally peer reviewed.

References

1. Angus, D.C., et al., *Epidemiology of severe sepsis in the United States: analysis of incidence, outcome, and associated costs of care.* Crit Care Med, 2001. **29**(7): p. 1303-10.
2. Hu, L., et al., *The incidence, risk factors and outcomes of acute kidney injury in critically ill patients undergoing emergency surgery: a prospective observational study.* BMC Nephrol, 2022. **23**(1): p. 42.
3. Chao, J., et al., *Detection of Early Cytokine Storm in Patients with Septic Shock After Abdominal Surgery.* J Transl Int Med, 2020. **8**(2): p. 91-98.
4. Cohen, J., et al., *Sepsis: a roadmap for future research.* Lancet Infect Dis, 2015. **15**(5): p. 581-614.
5. Adhikari, N.K., et al., *Critical care and the global burden of critical illness in adults.* Lancet, 2010. **376**(9749): p. 1339-46.
6. Jiang, W., et al., *ADJunctive Ulinastatin in Sepsis Treatment in China (ADJUST study): study protocol for a randomized controlled trial.* Trials, 2018. **19**(1): p. 133.

For peer review only

7. Dellinger, R.P., et al., *Surviving Sepsis Campaign: international guidelines for management of severe sepsis and septic shock, 2012.* Intensive Care Med, 2013. **39**(2): p. 165-228.
8. Dellinger, R.P., et al., *Surviving Sepsis Campaign: international guidelines for management of severe sepsis and septic shock: 2008.* Crit Care Med, 2008. **36**(1): p. 296-327.
9. Hollenberg, S.M., et al., *Practice parameters for hemodynamic support of sepsis in adult patients: 2004 update.* Crit Care Med, 2004. **32**(9): p. 1928-48.
10. Rehn, M., et al., *Surviving sepsis campaign: International guidelines for management of sepsis and septic shock in adults 2021 - endorsement by the Scandinavian society of anaesthesiology and intensive care medicine.* Acta Anaesthesiol Scand, 2022. **66**(5): p. 634-635.
11. Hotchkiss, R.S. and I.E. Karl, *The pathophysiology and treatment of sepsis.* N Engl J Med, 2003. **348**(2): p. 138-50.
12. Hotchkiss, R.S., G. Monneret, and D. Payen, *Sepsis-induced immunosuppression: from cellular dysfunctions to immunotherapy.* Nat Rev Immunol, 2013. **13**(12): p. 862-74.
13. Hotchkiss, R.S. and S. Opal, *Immunotherapy for sepsis--a new approach against an ancient foe.* N Engl J Med, 2010. **363**(1): p. 87-9.
14. Ou, P., H.J. Tritschler, and S.P. Wolff, *Thioctic (lipoic) acid: a therapeutic metal-chelating antioxidant?* Biochem Pharmacol, 1995. **50**(1): p. 123-6.
15. Rochette, L., et al., *Direct and indirect antioxidant properties of α-lipoic acid and therapeutic potential.* Molecular Nutrition & Food Research, 2013. **57**(1): p. 114-125.
16. Shay, K.P., et al., *Alpha-lipoic acid as a dietary supplement: molecular mechanisms and therapeutic potential.* Biochim Biophys Acta, 2009. **1790**(10): p. 1149-60.
17. Zhang, W.J., et al., *Alpha-lipoic acid attenuates LPS-induced inflammatory responses by activating the phosphoinositide 3-kinase/Akt signaling pathway.* Proc Natl Acad Sci U S A, 2007. **104**(10): p. 4077-82.
18. Demarco, V.G., et al., *alpha-lipoic acid inhibits endotoxin-stimulated expression of iNOS and nitric oxide independent of the heat shock response in RAW 264.7 cells.* Free Radic Res, 2004. **38**(7): p. 675-82.
19. Della Giustina, A., et al., *Alpha-lipoic acid attenuates acute neuroinflammation and long-term cognitive impairment after polymicrobial sepsis.* Neurochem Int, 2017. **108**: p. 436-447.
20. Petronilho, F., et al., *Alpha-Lipoic Acid Attenuates Oxidative Damage in Organs After Sepsis.* Inflammation, 2016. **39**(1): p. 357-365.
21. Li, G., et al., *Alpha-lipoic acid exerts anti-inflammatory effects on lipopolysaccharide-stimulated rat mesangial cells via inhibition of nuclear factor kappa B (NF-κB) signaling pathway.* Inflammation, 2015. **38**(2): p. 510-9.
22. Ziegler, D., *Thioctic acid for patients with symptomatic diabetic polyneuropathy: a critical review.* Treat Endocrinol, 2004. **3**(3): p. 173-89.

For peer review only

23. Melhem, A., et al., *Treatment of chronic hepatitis C virus infection via antioxidants: results of a phase I clinical trial.* J Clin Gastroenterol, 2005. **39**(8): p. 737-42.
24. Sola, S., et al., *Irbesartan and lipoic acid improve endothelial function and reduce markers of inflammation in the metabolic syndrome: results of the Irbesartan and Lipoic Acid in Endothelial Dysfunction (ISLAND) study.* Circulation, 2005. **111**(3): p. 343-8.
25. Cho, Y.S., et al., *alpha-Lipoic acid inhibits airway inflammation and hyperresponsiveness in a mouse model of asthma.* J Allergy Clin Immunol, 2004. **114**(2): p. 429-35.
26. Jesudason, E.P., et al., *The protective role of DL-alpha-lipoic acid in the oxidative vulnerability triggered by Abeta-amyloid vaccination in mice.* Mol Cell Biochem, 2005. **270**(1-2): p. 29-37.
27. Kim, N.W., et al., *Adjunctive α-lipoic acid reduces weight gain compared with placebo at 12 weeks in schizophrenic patients treated with atypical antipsychotics: a double-blind randomized placebo-controlled study.* Int Clin Psychopharmacol, 2016. **31**(5): p. 265-74.
28. Li, N., et al., *Effects of oral α-lipoic acid administration on body weight in overweight or obese subjects: a crossover randomized, double-blind, placebo-controlled trial.* Clin Endocrinol (Oxf), 2017. **86**(5): p. 680-687.
29. Khalili, M., et al., *Effect of lipoic acid consumption on oxidative stress among multiple sclerosis patients: a randomized controlled clinical trial.* Nutr Neurosci, 2014. **17**(1): p. 16-20.
30. Haghighian, H.K., et al., *Randomized, triple-blind, placebo-controlled clinical trial examining the effects of alpha-lipoic acid supplement on the spermatogram and seminal oxidative stress in infertile men.* Fertil Steril, 2015. **104**(2): p. 318-24.
31. Ametov, A.S., et al., *The sensory symptoms of diabetic polyneuropathy are improved with alpha-lipoic acid: the SYDNEY trial.* Diabetes Care, 2003. **26**(3): p. 770-6.
32. Garcia-Alcala, H., et al., *Treatment with α-Lipoic Acid over 16 Weeks in Type 2 Diabetic Patients with Symptomatic Polyneuropathy Who Responded to Initial 4-Week High-Dose Loading.* J Diabetes Res, 2015. **2015**: p. 189857.
33. Shen, H.H., et al., *Alpha-lipoic acid prevents endotoxic shock and multiple organ dysfunction syndrome induced by endotoxemia in rats.* Shock, 2015. **43**(4): p. 405-11.
34. Cadirci, E., et al., *Alpha-lipoic acid as a potential target for the treatment of lung injury caused by cecal ligation and puncture-induced sepsis model in rats.* Shock, 2010. **33**(5): p. 479-84.
35. Chictr.org.cn. *Lipoic acid injection in the treatment of adult patients with sepsis and septic shock: a prospective, multicenter, single blind, randomized, placebo-controlled clinical trial* 2021 [cited 2023 02/14]; Available from: https://www.chictr.org.cn/showprojEN.html?proj=62629.
36. Chan, A.W., et al., *SPIRIT 2013 explanation and elaboration: guidance for protocols of clinical trials.* BMJ, 2013. **346**: p. e7586.

For peer review only

37. Shankar-Hari, M., et al., *Developing a New Definition and Assessing New Clinical Criteria for Septic Shock: For the Third International Consensus Definitions for Sepsis and Septic Shock (Sepsis-3).* JAMA, 2016. **315**(8): p. 775-87.
38. Hejazi, N., et al., *The Beneficial Effects of α-Lipoic Acid in Critically Ill Patients: A Prospective, Randomized, Double-Blind, Placebo-Controlled Trial.* Asian Journal of Anesthesiology, 2018. **56**(2): p. 45-55.
39. Qu, R., et al., *C-reactive protein concentration as a risk predictor of mortality in intensive care unit: a multicenter, prospective, observational study.* BMC Anesthesiol, 2020. **20**(1): p. 292.
40. Mghamba, J.M., et al., *Developing a multisectoral National Action Plan for Health Security (NAPHS) to implement the International Health Regulations (IHR 2005) in Tanzania.* BMJ Glob Health, 2018. **3**(2): p. e000600.
41. Ozes, O.N., et al., *NF-kappaB activation by tumour necrosis factor requires the Akt serine-threonine kinase.* Nature, 1999. **401**(6748): p. 82-5.
42. Thomas, K.W., et al., *Respiratory syncytial virus inhibits apoptosis and induces NF-kappa B activity through a phosphatidylinositol 3-kinase-dependent pathway.* J Biol Chem, 2002. **277**(1): p. 492-501.
43. Madrid, L.V., et al., *Akt stimulates the transactivation potential of the RelA/p65 Subunit of NF-kappa B through utilization of the Ikappa B kinase and activation of the mitogen-activated protein kinase p38.* J Biol Chem, 2001. **276**(22): p. 18934-40.
44. Guha, M. and N. Mackman, *The phosphatidylinositol 3-kinase-Akt pathway limits lipopolysaccharide activation of signaling pathways and expression of inflammatory mediators in human monocytic cells.* J Biol Chem, 2002. **277**(35): p. 32124-32.
45. Kim, I., et al., *Angiopoietin-1 negatively regulates expression and activity of tissue factor in endothelial cells.* FASEB J, 2002. **16**(1): p. 126-8.
46. Bommhardt, U., et al., *Akt decreases lymphocyte apoptosis and improves survival in sepsis.* J Immunol, 2004. **172**(12): p. 7583-91.
47. Schabbauer, G., et al., *PI3K-Akt pathway suppresses coagulation and inflammation in endotoxemic mice.* Arterioscler Thromb Vasc Biol, 2004. **24**(10): p. 1963-9.
48. Williams, D.L., et al., *Modulation of the phosphoinositide 3-kinase pathway alters innate resistance to polymicrobial sepsis.* J Immunol, 2004. **172**(1): p. 449-56.
49. Rajaram, M.V., et al., *Akt/Protein kinase B modulates macrophage inflammatory response to Francisella infection and confers a survival advantage in mice.* J Immunol, 2006. **177**(9): p. 6317-24.
50. Fukao, T. and S. Koyasu, *PI3K and negative regulation of TLR signaling.* Trends Immunol, 2003. **24**(7): p. 358-63.
51. Martin, M., et al., *Toll-like receptor-mediated cytokine production is differentially regulated by glycogen synthase kinase 3.* Nat Immunol, 2005. **6**(8): p. 777-84.

For peer review only

52. Matsuda, N., et al., *Phosphorylation of endothelial nitric-oxide synthase is diminished in mesenteric arteries from septic rabbits depending on the altered phosphatidylinositol 3-kinase/Akt pathway: reversal effect of fluvastatin therapy.* J Pharmacol Exp Ther, 2006. **319**(3): p. 1348-54.
53. Ong, E., et al., *Role of phosphatidylinositol 3-kinase-gamma in mediating lung neutrophil sequestration and vascular injury induced by E. coli sepsis.* Am J Physiol Lung Cell Mol Physiol, 2005. **289**(6): p. L1094-103.
54. Collins, T., et al., *Transcriptional regulation of endothelial cell adhesion molecules: NF-kappa B and cytokine-inducible enhancers.* FASEB J, 1995. **9**(10): p. 899-909.
55. Karin, M., Z. Liu, and E. Zandi, *AP-1 function and regulation.* Curr Opin Cell Biol, 1997. **9**(2): p. 240-6.
56. Liu, H., et al., *Redox-dependent transcriptional regulation.* Circ Res, 2005. **97**(10): p. 967-74.
57. Liu, S.F. and A.B. Malik, *NF-kappa B activation as a pathological mechanism of septic shock and inflammation.* Am J Physiol Lung Cell Mol Physiol, 2006. **290**(4): p. L622-L645.
58. Böhrer, H., et al., *Role of NFkappaB in the mortality of sepsis.* J Clin Invest, 1997. **100**(5): p. 972-85.
59. Arnalich, F., et al., *Predictive value of nuclear factor kappaB activity and plasma cytokine levels in patients with sepsis.* Infect Immun, 2000. **68**(4): p. 1942-5.
60. Miliaraki, M., et al., *Oxidant/Antioxidant Status Is Impaired in Sepsis and Is Related to Anti-Apoptotic, Inflammatory, and Innate Immunity Alterations.* Antioxidants (Basel), 2022. **11**(2).
61. Crimi, E., et al., *Role of oxidative stress in experimental sepsis and multisystem organ dysfunction.* Free Radic Res, 2006. **40**(7): p. 665-72.
62. Joffre, J. and J. Hellman, *Oxidative Stress and Endothelial Dysfunction in Sepsis and Acute Inflammation.* Antioxid Redox Signal, 2021. **35**(15): p. 1291-1307.
63. Galley, H.F., *Oxidative stress and mitochondrial dysfunction in sepsis.* Br J Anaesth, 2011. **107**(1): p. 57-64.
64. Goode, H.F. and N.R. Webster, *Free radicals and antioxidants in sepsis.* Crit Care Med, 1993. **21**(11): p. 1770-6.

For peer review only

Supplementary Table 1 Trial schedule of the LAS study

Timepoint	Baseline	[a]Interventions									Termination	Follow-up period
	[b]D0	D1	D2	D3	D4	D5	D6	D7	D8-10	D…	D28	D…
Eligibility screening	×											
Informed consent obtaining	×											
Randomization	×											
Medical history collection	×											
Population data collection	×											
Baseline data collection	×											
Formulation of drug administration plan	×											
Evaluation of drug-related adverse reactions		×	×	×	×	×	×	×	×			
Evaluate serious adverse events		×	×	×	×	×	×	×	×			

For peer review only

Timepoint	Baseline	[a]Interventions									Termination	Follow-up period
	[b]D0	D1	D2	D3	D4	D5	D6	D7	D8-10	D…	D28	D…
Vital signs	×	×	×	×	×	×	×	×			×	
SOFA/APACHEIIscore	×	×		×		×		×				
[c]Need and duration of life support treatment		×	×	×	×	×	×	×	×	×	×	
[d]Levels of inflammatory mediators	×	×		×		×		×				
Arterial lactic acid level	×	×		×		×		×				
Transfer out of ICU		×	×	×	×	×	×	×	×	×	×	×
ICU death time		×	×	×	×	×	×	×	×	×	×	×
AKI occurrence time		×	×	×	×	×	×	×	×	×	×	×
Hospital death time		×	×	×	×	×	×	×	×	×	×	×
Discharge time		×	×	×	×	×	×	×	×	×	×	×

Eligibility screening includes diagnosis, inclusion/exclusion criteria, urine pregnancy test for female, demographics, medical history and physical examination. [a]Intervention means successful enrollment; [b]D means day from enrollment; [c]Life support therapy: vasopressor, invasive

mechanical ventilation and continuous renal replacement therapy; [d]Inflammatory mediators: interleukin -2(IL-2), IL-4, IL-6, IL-10, tumor necrosis factor-α (TNF-α), interferon-γ (IFN-γ), procalcitonin (PCT) and high-sensitivity C-reactive protein (hs-CRP); SOFA, Sequential Organ Failure Assessment score; APECHE Ⅱ, Acute Physiology and Chronic Health Evaluation; AKI, Acute Kidney Injury.

For peer review only

知情同意书▪知情告知页

尊敬的先生/女士：

您好！您（或您的家属）当前所患的是☐脓毒症☐脓毒性休克。我们将邀请您（或您的家属）参加一项已上市药品治疗此疾病的临床研究：硫辛酸注射液治疗脓毒症及脓毒性休克成人患者的有效性及安全性的前瞻性、多中心、单盲、随机、安慰剂对照临床研究。该研究由茂名市人民医院、广东省人民医院发起。为了保证您的权益，本研究的研究方案和知情同意书均已获得伦理委员会的批准（编号：________________）。在同意参加这项研究之前，阅读并理解这份知情同意书对您来说十分重要。这份文件向您阐述了本研究的研究目的、步骤、可能给您带来的受益和风险等注意事项。请您仔细阅读，如有任何疑问请向负责该项研究的研究者提出。您参加本项研究是自愿的，并且可以在任何时间阶段退出研究。如果您决定参加这项研究，您将获得一份由双方签名的知情同意书副本。

一、研究背景与目的

脓毒症是机体对感染的反应失调而导致危及生命的器官功能障碍，脓毒性休克是指脓毒症合并出现严重的循环障碍和细胞代谢紊乱，是感染、烧/创伤、休克等急危重患者的严重并发症。脓毒症与脓毒性休克病死率高，大幅增加患者及家属经济负担。随着我国危重病监护救治技术的进步，脓毒症患者病死率虽然已显著下降，但仍高达 20~30%。针对脓毒症的病因治疗临床尚无特效药物。

研究表明，硫辛酸及其代谢产物是体内天然的生物抗氧化剂，鉴于氧化应激介导的细胞损伤是脓毒症重要病理生理学基础，通过捕捉体内自由基，从而抑制体内过度的炎症反应，改善脓毒症及脓毒性休克的治疗是可行的。我们拟在确诊脓毒症或脓毒性休克患者中应用硫辛酸治疗，探讨硫辛酸降低脓毒症及脓毒性休克患者病死率的有效性和安全性，以期降低患者病死率、提高救治成功率、缩短住院天数并改善改善预后。

二、研究内容和过程

本项研究采用前瞻性、多中心、单盲、随机、安慰剂对照临床研究设计，如果您符合本研究的入组标准，您将在脓毒症/脓毒性休克常规治疗（依据《拯救脓毒症和脓毒性休克运动指南》）或在常规治疗的基础上应用硫辛酸治疗，您分配在两种方案（硫辛酸组和安慰剂组）中接受治疗的概率是相同的。

本研究将记录您的个人情况和与疾病相关的临床资料：包括病史；常规医学检查（如血尿常规、肝肾功能、凝血功能、生化检查、血气分析、炎性因子和炎症标记物、影像学检查、

For peer review only - http://bmjopen.bmj.com/site/about/guidelines.xhtml

医学评分等）；为了客观评价病情变化，还将详细询问并记录您住院期间每天的病情变化，出院后随访至您入组后第 28 天。

以上治疗措施和医学检查项目，均为脓毒症和脓毒性休克诊断和治疗所必须的常规项目。本研究未设计超出常规诊断和治疗所必须的特殊检查和治疗项目，未增加患者的额外医疗费用。

三、参与本研究可能的受益

您和社会将可能从本研究中受益。此种受益包括您的病情有可能获得改善，减少器官损害的程度，硫辛酸免费用药，以及本研究可能帮助患有相似病情的其他患者。

无论您是否参加本研究，治疗与相关医学检查都会按本研究的常规方案进行。因此，参加本研究不会额外增加您的医疗负担；并且对您的病情观察和治疗将会更加全面、有利。

四、参与本研究可能的风险与不适

静脉滴注硫辛酸一般可能出现的不良反应有：例如头胀、呼吸困难、抽搐、复视、紫癜、出血倾向、过敏等。反应大多较轻且发生率较低，停药后可自行消失。

五、研究的保密性

如果您决定参加本项研究，您参加研究及在研究中的个人资料均属保密。您的血/尿等标本将以研究编号数字而非您的姓名加以标识。可以识别您身份的信息将不会透露给研究小组以外的成员，除非获得您的许可。所有的研究成员都被要求对您的身份保密。您的档案将保存在有锁的档案柜中，仅供研究人员查阅。为确保研究按照规定进行，必要时，政府管理部门或伦理委员会的成员按规定可以在研究单位查阅您的个人资料。这项研究结果发表时，将不会披露您个人的任何资料。

六、您的权利与义务

参加本研究期间需要您配合研究方案要求，接受研究医生对疾病预后的随访。

您可以选择不参加本项研究，或者在任何时候通知研究者要求退出研究，您做出的决定绝不影响您和医务人员的关系，您的任何医疗待遇、权益和医学关注不会因此受到影响，不会受到任何不公平的对待和惩罚。如果您决定退出研究，请您务必与您的研究医生联络，如果您在研究期间终止治疗和检查，本着对您的健康负责的目的，研究医生可能会询问一些与您健康相关的问题，还有可能要求您进行一些检查。

如果您需要其它治疗，或者没有遵循研究计划，或者您的主管研究医生认为您继续参与本研究不符合您的最大利益，研究医生可以让您退出研究；如果您在使用研究药物后出现不

For peer review only - http://bmjopen.bmj.com/site/about/guidelines.xhtml

适，或者安全风险，研究医生可能会在未征得您同意的情况下让您退出研究，研究医生将会与您讨论您退出研究后的相关事宜。

您可随时了解与本研究有关的信息资料和研究进展，如果您有与本研究有关的问题，或您在研究过程中发生了任何不适与损伤，或有关于本项研究参加者权益方面的问题您可以跟研究者联系。如果您对您作为研究参与者的权利有任何问题，您可以联系伦理委员会人员，电话：15915280880 。

致谢

医学科学的发展和进步离不开临床研究，您的参与将为医学科学进步做出贡献，对该疾病诊疗的研究与探索做出贡献。作为此项研究的研究者和申办者，我们将时刻铭记您的贡献，并对您表示最诚挚的感谢。

For peer review only - http://bmjopen.bmj.com/site/about/guidelines.xhtml

知情同意书 ▪ 同意签字页

在签署这份知情同意书前，我已经阅读上述信息，并且理解该项目的目的以及参加该项目可能带来的潜在利益和风险。我确认已经经过充分的考虑，也有机会对研究程序和研究药物提出疑问，并且所有的疑问均已得到令我满意的解答。

我同意研究医生收集和处理我的信息，包括与我健康有关的信息。我同意我的信息（个人信息除外）由研究单位处理。如果我决定退出本研究，我同意在此之前收集的信息仍可以被继续处理。

我有权在任何时候得到咨询服务，并有权决定在任何时候退出本研究计划而不会受到任何不利影响，不会因此丧失任何合法权利。我自愿签署这份知情同意书，并自愿参加此研究项目，会与研究者全面合作。我已得到此份文件的副本。

受试者

姓名(正楷) ____________________ 签名:__________________________

联系电话: _____________________ 日期: _____年____月____日

法定代理人/ 法定监护人

姓名(正楷) _________________ 签名:____________ 与受试者关系：_____________

联系电话: __________________ 日期: _____年____月____日

公正的见证人声明

我确认，同意书中的信息被进行了准确的解释，已经被患者和/或患者的法定代理人理解，同意意见是由患者和/或患者的法定代理人自愿提供的。

姓名(正楷) ____________________ 签名:__________________________

联系电话: _____________________ 日期: _____年____月____日

注：如果患者、法定监护人或法定代理人不能阅读及签署同意书（例如重度视力受损、诵读困难或不识字），必须有至少 1 名公正的见证人。公正的见证人在讨论知情同意书的整个过程中都必须在场。

我已准确地将知情同意书内容告知受试者并对受试者的提问进行了解答，受试者自愿参加本项临床研究。

研究者签名：_____________ 联系电话：________________

日期: ____年____月____日

For peer review only - http://bmjopen.bmj.com/site/about/guidelines.xhtml

Reporting checklist for protocol of a clinical trial.

Based on the SPIRIT guidelines.

Instructions to authors

Complete this checklist by entering the page numbers from your manuscript where readers will find each of the items listed below.

Your article may not currently address all the items on the checklist. Please modify your text to include the missing information. If you are certain that an item does not apply, please write "n/a" and provide a short explanation.

Upload your completed checklist as an extra file when you submit to a journal.

In your methods section, say that you used the SPIRIT reporting guidelines, and cite them as:

Chan A-W, Tetzlaff JM, Gøtzsche PC, Altman DG, Mann H, Berlin J, Dickersin K, Hróbjartsson A, Schulz KF, Parulekar WR, Krleža-Jerić K, Laupacis A, Moher D. SPIRIT 2013 Explanation and Elaboration: Guidance for protocols of clinical trials. BMJ. 2013;346:e7586

		Reporting Item	Page Number
**Administrative information**			
Title	#1	Descriptive title identifying the study design, population, interventions, and, if applicable, trial acronym	1
Trial registration	#2a	Trial identifier and registry name. If not yet registered, name of intended registry	3
Trial registration: data set	#2b	All items from the World Health Organization Trial Registration Data Set	7
Protocol version	#3	Date and version identifier	n/a, no major amendments made
Funding	#4	Sources and types of financial, material, and other support	20

For peer review only

Roles and responsibilities: contributorship	#5a	Names, affiliations, and roles of protocol contributors	19
Roles and responsibilities: sponsor contact information	#5b	Name and contact information for the trial sponsor	n/a, it is a investigator initiated trial
Roles and responsibilities: sponsor and funder	#5c	Role of study sponsor and funders, if any, in study design; collection, management, analysis, and interpretation of data; writing of the report; and the decision to submit the report for publication, including whether they will have ultimate authority over any of these activities	20
Roles and responsibilities: committees	#5d	Composition, roles, and responsibilities of the coordinating centre, steering committee, endpoint adjudication committee, data management team, and other individuals or groups overseeing the trial, if applicable (see Item 21a for data monitoring committee)	13
**Introduction**			
Background and rationale	#6a	Description of research question and justification for undertaking the trial, including summary of relevant studies (published and unpublished) examining benefits and harms for each intervention	5-6
Background and rationale: choice of comparators	#6b	Explanation for choice of comparators	5-6
Objectives	#7	Specific objectives or hypotheses	9-11
Trial design	#8	Description of trial design including type of trial (eg, parallel group, crossover, factorial, single group), allocation ratio, and framework (eg, superiority, equivalence, non-inferiority, exploratory)	7, 12-13
**Methods: Participants,**			

For peer review only

**interventions, and outcomes**			
Study setting	#9	Description of study settings (eg, community clinic, academic hospital) and list of countries where data will be collected. Reference to where list of study sites can be obtained	7-8
Eligibility criteria	#10	Inclusion and exclusion criteria for participants. If applicable, eligibility criteria for study centres and individuals who will perform the interventions (eg, surgeons, psychotherapists)	9
Interventions: description	#11a	Interventions for each group with sufficient detail to allow replication, including how and when they will be administered	10
Interventions: modifications	#11b	Criteria for discontinuing or modifying allocated interventions for a given trial participant (eg, drug dose change in response to harms, participant request, or improving / worsening disease)	11
Interventions: adherance	#11c	Strategies to improve adherence to intervention protocols, and any procedures for monitoring adherence (eg, drug tablet return; laboratory tests)	n/a, adherence is not the issue in the settings of intensive care unit
Interventions: concomitant care	#11d	Relevant concomitant care and interventions that are permitted or prohibited during the trial	10
Outcomes	#12	Primary, secondary, and other outcomes, including the specific measurement variable (eg, systolic blood pressure), analysis metric (eg, change from baseline, final value, time to event), method of aggregation (eg, median, proportion), and time point for each outcome. Explanation of the clinical relevance of chosen efficacy and harm outcomes is strongly recommended	10-11
Participant timeline	#13	Time schedule of enrolment, interventions (including any run-ins and washouts), assessments, and visits for participants. A schematic diagram is highly	27-29

For peer review only

		recommended (see Figure)	
Sample size	#14	Estimated number of participants needed to achieve study objectives and how it was determined, including clinical and statistical assumptions supporting any sample size calculations	11-12
Recruitment	#15	Strategies for achieving adequate participant enrolment to reach target sample size	12
**Methods: Assignment of interventions (for controlled trials)**			
Allocation: sequence generation	#16a	Method of generating the allocation sequence (eg, computer-generated random numbers), and list of any factors for stratification. To reduce predictability of a random sequence, details of any planned restriction (eg, blocking) should be provided in a separate document that is unavailable to those who enrol participants or assign interventions	12-13
Allocation concealment mechanism	#16b	Mechanism of implementing the allocation sequence (eg, central telephone; sequentially numbered, opaque, sealed envelopes), describing any steps to conceal the sequence until interventions are assigned	12-13
Allocation: implementation	#16c	Who will generate the allocation sequence, who will enrol participants, and who will assign participants to interventions	12-13
Blinding (masking)	#17a	Who will be blinded after assignment to interventions (eg, trial participants, care providers, outcome assessors, data analysts), and how	13
Blinding (masking): emergency unblinding	#17b	If blinded, circumstances under which unblinding is permissible, and procedure for revealing a participant's allocated intervention during the trial	13
**Methods: Data collection, management, and analysis**			

For peer review only

Data collection plan	#18a	Plans for assessment and collection of outcome, baseline, and other trial data, including any related processes to promote data quality (eg, duplicate measurements, training of assessors) and a description of study instruments (eg, questionnaires, laboratory tests) along with their reliability and validity, if known. Reference to where data collection forms can be found, if not in the protocol	11
Data collection plan: retention	#18b	Plans to promote participant retention and complete follow-up, including list of any outcome data to be collected for participants who discontinue or deviate from intervention protocols	n/a, the follow-up window in our trial is a maximum of 28 days, which sees few lost to follow-up
Data management	#19	Plans for data entry, coding, security, and storage, including any related processes to promote data quality (eg, double data entry; range checks for data values). Reference to where details of data management procedures can be found, if not in the protocol	13
Statistics: outcomes	#20a	Statistical methods for analysing primary and secondary outcomes. Reference to where other details of the statistical analysis plan can be found, if not in the protocol	14-15
Statistics: additional analyses	#20b	Methods for any additional analyses (eg, subgroup and adjusted analyses)	n/a, subgroup and adjusted analyses is not planned
Statistics: analysis population and missing data	#20c	Definition of analysis population relating to protocol non-adherence (eg, as randomised analysis), and any statistical methods to handle missing data (eg, multiple imputation)	14

Methods: Monitoring

For peer review only

Data monitoring: formal committee	#21a	Composition of data monitoring committee (DMC); summary of its role and reporting structure; statement of whether it is independent from the sponsor and competing interests; and reference to where further details about its charter can be found, if not in the protocol. Alternatively, an explanation of why a DMC is not needed	13
Data monitoring: interim analysis	#21b	Description of any interim analyses and stopping guidelines, including who will have access to these interim results and make the final decision to terminate the trial	15
Harms	#22	Plans for collecting, assessing, reporting, and managing solicited and spontaneously reported adverse events and other unintended effects of trial interventions or trial conduct	10-11
Auditing	#23	Frequency and procedures for auditing trial conduct, if any, and whether the process will be independent from investigators and the sponsor	13
**Ethics and dissemination**			
Research ethics approval	#24	Plans for seeking research ethics committee / institutional review board (REC / IRB) approval	7
Protocol amendments	#25	Plans for communicating important protocol modifications (eg, changes to eligibility criteria, outcomes, analyses) to relevant parties (eg, investigators, REC / IRBs, trial participants, trial registries, journals, regulators)	7
Consent or assent	#26a	Who will obtain informed consent or assent from potential trial participants or authorised surrogates, and how (see Item 32)	12
Consent or assent: ancillary studies	#26b	Additional consent provisions for collection and use of participant data and biological specimens in ancillary studies, if applicable	12
Confidentiality	#27	How personal information about potential and enrolled	13

For peer review only

		participants will be collected, shared, and maintained in order to protect confidentiality before, during, and after the trial	
Declaration of interests	#28	Financial and other competing interests for principal investigators for the overall trial and each study site	20
Data access	#29	Statement of who will have access to the final trial dataset, and disclosure of contractual agreements that limit such access for investigators	13
Ancillary and post trial care	#30	Provisions, if any, for ancillary and post-trial care, and for compensation to those who suffer harm from trial participation	15
Dissemination policy: trial results	#31a	Plans for investigators and sponsor to communicate trial results to participants, healthcare professionals, the public, and other relevant groups (eg, via publication, reporting in results databases, or other data sharing arrangements), including any publication restrictions	15
Dissemination policy: authorship	#31b	Authorship eligibility guidelines and any intended use of professional writers	n/a, all authorship will be determined by the principal investigator according actual contributions
Dissemination policy: reproducible research	#31c	Plans, if any, for granting public access to the full protocol, participant-level dataset, and statistical code	15
**Appendices**			
Informed consent materials	#32	Model consent form and other related documentation given to participants and authorised surrogates	12
Biological specimens	#33	Plans for collection, laboratory evaluation, and storage of biological specimens for genetic or molecular analysis in the current trial and for future use in	n/a, blood specimens will be

For peer review only

	ancillary studies, if applicable	simultaneously collected and tested in hospital clinical laboratory as routine

None The SPIRIT Explanation and Elaboration paper is distributed under the terms of the Creative Commons Attribution License CC-BY-NC. This checklist can be completed online using https://www.goodreports.org/, a tool made by the EQUATOR Network in collaboration with Penelope.ai

For peer review only
